# DNA methylation-based age acceleration observed in IDH wild-type glioblastoma is associated with better outcome—including in elderly patients

**DOI:** 10.1186/s40478-022-01344-5

**Published:** 2022-03-24

**Authors:** Pierre Bady, Christine Marosi, Michael Weller, Bjørn H. Grønberg, Henrik Schultz, Martin J. B. Taphoorn, Johanna M. M. Gijtenbeek, Martin J. van den Bent, Andreas von Deimling, Roger Stupp, Annika Malmström, Monika E. Hegi

**Affiliations:** 1 Swiss Institute of Bioinformatics (SIB), Lausanne, Switzerland; 2 Lausanne University Hopital and University of Lausanne, Chemin des Boveresses 155, CLE-C306, 1066 Epalinges, Switzerland; 3 Medical Oncology, University of Vienna, Vienna, Austria; 4 Department of Neurology, University Hospital and University of Zurich, Zurich, Switzerland; 5 Department of Clinical and Molecular Medicine, Norwegian University of Science and Technology and Department of Oncology, St. Olavs Hospital, Trondheim, Norway; 6 Aarhus University Hospital, Aarhus, Denmark; 7 Departments of Neurology, Leiden University Medical Center and Haaglanden Medical Center, Leiden and The Hague, Netherlands; 8 Department of Neurology, Radboud University Medical Centre, Nijmegen, Netherlands; 9 Brain Tumor Center, Erasmus MC Cancer Institute, Rotterdam, Netherlands; 10 Department of Neuropathology, Institute of Pathology, University of Heidelberg, and CCU Neuropathology, German Cancer Center (DKFZ), Heidelberg, Germany; 11 Malnati Brain Tumor Institute of the Lurie Comprehensive Cancer Center, Department of Neurological Surgery and Department of Neurology, Northwestern Medicine and Northwestern University, Chicago, IL USA; 12 Department of Biomedical and Clinical Sciences and Department of Advanced Home Care, Linköping University, Linköping, Sweden; 13 Service of Neurosurgery, Lausanne University Hospital (CHUV), University of Lausanne (UNIL), Lausanne, Switzerland; 14 Swiss Cancer Center Léman (SCCL), Lausanne, Switzerland

**Keywords:** DNA methylation age acceleration, Glioblastoma IDHwt, Survival, Methylome, Age

## Abstract

Elderly patients represent a growing proportion of individuals with glioblastoma, who however, are often excluded from clinical trials owing to poor expected prognosis. We aimed at identifying age-related molecular differences that would justify and guide distinct treatment decisions in elderly glioblastoma patients. The combined DNA methylome (450 k) of four IDH wild-type glioblastoma datasets, comprising two clinical trial cohorts, was interrogated for differences based on the patients’ age, DNA methylation (DNAm) age acceleration (DNAm age “Horvath-clock” minus patient age), DNA methylation-based tumor classification (Heidelberg), entropy, and functional methylation of DNA damage response (DDR) genes. Age dependent methylation included 19 CpGs (p-value ≤ 0.1, Bonferroni corrected), comprising a CpG located in the *ELOVL2* gene that is part of a 13-gene forensic age predictor. Most of the age related CpGs (n = 16) were also associated with age acceleration that itself was associated with a large number of CpGs (n = 50,551). Over 70% age acceleration-associated CpGs (n = 36,348) overlapped with those associated with the DNA methylation based tumor classification (n = 170,759). Gene set enrichment analysis identified associated pathways, providing insights into the biology of DNAm age acceleration and respective commonalities with glioblastoma classification. Functional methylation of several DDR genes, defined as correlation of methylation with gene expression (r ≤ −0.3), was associated with age acceleration (n = 8), tumor classification (n = 12), or both (n = 4), the latter including *MGMT*. DNAm age acceleration was significantly associated with better outcome in both clinical trial cohorts, whereof one comprised only elderly patients. Multivariate analysis included treatment (RT, RT/TMZ→TMZ; TMZ, RT), *MGMT* promoter methylation status, and interaction with treatment. In conclusion, DNA methylation features of age acceleration are an integrative part of the methylation-based tumor classification (RTK I, RTK II, MES), while patient age seems hardly reflected in the glioblastoma DNA methylome. We found no molecular evidence justifying other treatments in elderly patients, not owing to frailty or co-morbidities.

**Supplementary Information:**

The online version contains supplementary material available at 10.1186/s40478-022-01344-5.

## Introduction

Patients over the age of 70 represent an increasing fraction of individuals with glioblastoma (GBM) [40], yet elderly patients are often excluded from clinical trials due to the estimated poor prognosis and a short overall survival (6–9 months). Usually, a shorter duration radiation therapy schedule in association with temozolomide (TMZ) is favored for elderly patients aiming to shorten the treatment duration in view of the overall short life expectancy. Hypofractionated radiotherapy (15 × 2.66 Gy) has been shown clinically equivalent to standard 30 × 2 Gy fractionation [43], in combination with TMZ chemotherapy this regimen has been superior to hypofractionated radiotherapy (RT) alone [42]. In tumors with a methylated promoter of the O6-methyl-guanine methyl transferase (*MGMT*) gene, exclusive or initial TMZ chemotherapy alone may be an alternative to radiotherapy. Median overall survival remains unsatisfactory with treatment regimens. The choice of the treatment regimen depends on the patient’s biological rather than chronological age and frailty, and the tumor *MGMT* promoter methylation status [59]. Yet, a fraction of elderly patients have a more favorable outcome with disease control beyond 18–24 months. Little is known about the molecular make-up of GBM in older patients (7th and 8th decade of life) and whether it differs from a “general” GBM IDH wild-type (IDHwt) population. We have reported previously from the Nordic trial for elderly GBM patients that the *MGMT* promoter methylation status is not associated with patient age [33]. The predictive value of the *MGMT* status for benefit from TMZ is supported by the aforementioned trials. Expectedly, mutations in the isocitrate dehydrogenase gene 1 (*IDH1*), a hallmark of gliomas in young adults, are rare in the elderly [33, 60]. Moreover, no obvious GBM-specific genetic alterations have been associated with the age of the patients, once removing cases with IDH mutant (IDHmut) tumors that are recognized and classified as a distinct disease entity since 2016 [31]. Hence, systematic molecular characterization is required, in order to justify distinct treatment modalities not owing to frailty of the patients, or suggesting new, more adapted (targeted) approaches that can be tested in clinical trials in this elderly patient population.

The DNA methylome of cells is known to contain age related information reflected in age associated epigenetic changes. These are thought to arise from innate biological mechanisms, such as deterioration of epigenetic maintenance resulting in changes of DNA methylation over time that allowed the construction of accurate predictors of chronologic age. Such predictors are known as *epigenetic clocks* or *DNA methylation clocks* [12, 16, 21, 22, 62]. Furthermore, certain biological features or environmental factors have been associated with an *acceleration* of the DNA methylation age (DNAm age) in the blood, certain tissues, and cancer. This difference between chronologic age of the patient and estimated DNAm age is called DNAm age acceleration, and has been found associated with obesity, and smoking, but also Down syndrome or cancer, as reviewed by Horvath and Raj [23]. It has been proposed that biological age acceleration may hold the potential for disease specific biomarkers or frailty measures for individuals [5, 23, 34]. In addition, the tumor DNA methylome comprises information on cell of origin, plus tumor development related alterations that allow accurate tumor classification and identification of new tumor entities [9, 37]. Previous studies have reported on associations of tumor DNAm age with chronologic age of the patients in adult glioma and GBM [30, 65]. However, these studies included glioma of variable grades and subtypes, including IDHmut glioma that constitute an epigenetically and clinically distinct entity, associated with a glioma CpG island methylator phenotype (G-CIMP +) [10, 39, 51].

In this translational research study we aimed at identifying age-related molecular differences that may unravel novel therapeutic options and guide rational management of elderly GBM patients. To this end we set out to investigate the DNA methylome of adult IDHwt GBM for age related differences, including the patients’ age, DNAm age acceleration of the tumor, DNA methylation-based tumor classification (Heidelberg), and CpG methylation entropy. Further, we paid special attention to the potential implication of DNA damage response (DDR) genes, given the current standard of care, treating GBM patients with DNA damaging therapies. We stablished the methylome for two clinical trial cohorts treating newly diagnosed GBM patients [33, 48, 49], whereof one recruited elderly patients only [33]. The study included an additional two external GBM data sets [8, 9], and retained only tumors meeting the criteria of GBM grade 4 of the recent update of the WHO CNS5 classification 2021 [32].

## Material and methods

### Patient samples

Two sets of newly diagnosed GBM samples were obtained from patients treated in clinical trials according to pre-specified clinical criteria: from the Nordic trial (n = 116), and from the trial conducted by the European Organisation for Research and Treamtent of Cancer (EORTC) and the National Cancer Institute of Canada (NCIC) (EORTC 26,981/NCIC CE.3), pooled with the samples from the Lausanne Pilot clinical trial (LN-Pilot; Biobank of the Brain and Spine Tumor Center, BB_031_BBLBGT, of the Centre Hospitalier Universitaire Vaudois, CHUV, Lausanne, Switzerland) (n = 219). The constituted clinical trial cohorts were restricted to patients for whom enough frozen or paraffin embedded tissue was available, thus excluding cases with diagnostic biopsies only. The cohorts overlap largely with those for which the *MGMT* promoter methylation status was reported in the original trials [19, 20, 33]. The Nordic phase III trial recruited patients 60 years or older with a WHO performance score (PS) [58] of 2 or less, allowing PS 3, if it was due to neurological deficits. Patients were randomized to one of two regimens of RT (60 Gy in 30 fractions, or 34 G in 10 fractions) or standard dose TMZ (200 mg/m^2^, days 1–5 every 28 days) [33] (trial registration number ISRCTN81470623). The LN-Pilot trial and the EORTC 26,981/NCIC CE.3 trial (NCT00006353) recruited patients between the ages of 18 and 70, and a WHO PS of 2 or less [48, 50]. Patients in the uncontrolled phase II LN-Pilot trial received the TMZ/RT→TMZ standard regimen (the current standard of care), and patients in the pivotal trial EORTC 26,981/NCIC CE.3 were randomized to RT (60 Gy in 30 fractions) or TMZ/RT→TMZ. Patients signed informed consent for translational research according to institutional and international guidelines and regulations.

### DNA methylation analysis

For genome-wide DNA methylation analysis DNA was isolated from macro-dissected tumor tissue (frozen samples: DNeasy Blood & Tissue Kit, Qiagen; formalin fixed paraffin embedded (FFPE) samples: EX-WAX™ Paraffin-embedded DNA Extraction Kit, S4530; Merck KGAa) and quantified (Quant-iT™ PicoGreen® dsDNA Assay Kit, #P7589, Life Technologies). DNA samples were analyzed on the Human Methylation 450 K BeadChip (Illumina, San Diego CA, USA) at the Genomics platform of the University of Geneva. FFPE-derived tumor DNA samples were processed after passing a PCR-based quality control (Infinium HD FFPE QC Assay Protocol). DNA samples were subjected to bisulfite treatment (EZ DNA Methylation-Gold™ Kit, Zymo Research) as previously described, and FFPE samples were analyzed in separate batches after pretreatment with the restauration kit as recommended (llumina) [3, 26].

### Data availability of own datasets

The datasets from the trial cohorts are available at the Gene Expression Omnibus database (GEO) (http://www.ncbi.nlm.nih.gov/geo/) under the accession numbers GSE195684 for the Nordic trial samples, and the samples from the LN-Pilot trial and the EORTC 26,981/NCIC CE.3 trial are available at GSE195640, or GSE60274. The latter comprises data from a subset of GBM samples of the EORTC-NCIC & Pilot trials and 5 non-tumoral brain tissue samples that we have previously published [26]. Methylation data from an additional 5 non-tumoral brain tissues used, is available under GSE104293 [3].

### External datasets

External datasets comprised the GBM dataset from The Cancer Genome Atlas (TCGA,) for which RNA-seq and HM-450 k data, and corresponding annotations [8] (TCGA; n = 113) were used. The dataset is available in the database of Genotypes and Phenotypes (dbGaP), dbGaP accession number phs000178.v9.p8; http://cancergenome.nih.gov. The GBM dataset with HM-450 k data from the Deutsches Krebsforschungszentrum (DKFZ) [9] (n = 235) was downloaded from GEO under the accession number GSE109381.

### Preprocessing DNA methylation data

The CpG probes with detection p-values > 0.01, located on the sex chromosomes, or in SNPs were removed from each dataset. The functional normalization [13] for Illumina 450 k arrays includes noob (normal-exponential out-of-band) background correction, dye-correction (chemistry I vs II) and RUV-2 step (removing unwanted variation) based on control probes. This normalization was performed by the function preprocessFunnorm from the R package minfi. DNA methylation was summarized by M-values [11]. The ComBat procedure [24] based on common CpG probes was used to aggregate the four datasets to limit experimental variation and batch effects across the four datasets.

### Copy number variation

Copy Number status for each marker was assessed using the combined intensities for methylated and unmethylated signals and circular binary segmentation to detect copy number aberration (CNA) events as previously described [2]. The homozygous deletion status (HD) of *CDKN2A* was determined using copy number probe means (CpGs) located in the *CDKN2A* gene and applying a mixture model [55].

### Additional metrics based on DNA methylation

#### Sample purity

The tumor purity (HMpurity) of each sample was estimated as previously proposed [3] using the GBM TCGA datasets.

#### MGMT promoter methylation status

The DNA methylation status of the *MGMT* promoter and the *MGMT* score (logit-transformed probability) were determined using the MGMT-STP27 regression model based on HM-450 k data [2, 4]. In brief, the M-values of the methylation probes cg12434587and cg12981137 were used as input into the logistic regression model (MGMT-STP27). A cut-off of 0.358 is used for classification into *MGMT* methylated and unmethylated promoter status, respectively.

### Molecular subtypes

The G-CIMP status was determined by unsupervised clustering (Ward’s algorithm with Euclidean distance) as previously reported [3] and served as approximation for the IDH mutation status, as this information was not available for all samples in any of the datasets. All G-CIMP + samples were removed. Molecular subtypes were obtained using the classification procedure of central nervous system tumors based on the analysis of DNA methylation patterns [9] (version v11b4, www.molecularneuropathology.org). For the main analyses of this study, we considered only GBM classified as mesenchymal (GBM_MES), RTK I (GBM_RTK_I) or RTK II (GBM_RTK_II).

### DNAage and age acceleration

DNA methylation-based estimates of age (DNAm age) were calculated using ElasticNet regression [66] using 353 CpG sites selected by the Horvath clock [21, 23]. The DNA methylation data were calibrated before the computation of DNAm age as recommended [21]. The metric called Age Acceleration (**Accel**) was obtained by subtraction of chronological age (**age**) from DNA methylation age (**DNAm age**). For subsequent analysis, 306 clock probes were conserved after filtering and aggregation of the four datasets (detection p-values > 0.01).

### DNA methylation entropy (HME)

Estimation of the DNA methylation entropy (HME) is given by the normalized Shannon entropy [46] adapted for methylation fraction (p) given by Beta values [16]. For the i^th^ methylation marker, two states were possible: unmethylated (*1 − pi*) or methylated (*pi)* and the maximal entropy is given by log(2). The DNA methylation Entropy (HME) for N methylation markers is computed as follow:

$$H{ME}_{m}=\frac{{\sum }_{i}\left[{p}_{u}\times log\left({p}_{u}\right)+{p}_{m}\times log\left({p}_{m}\right)\right]}{N\times log\left(1/2\right)}$$

The HME metrics were defined for all CpGs (global HM-entropy) and for the 12 strata constituted by the Island regions (CpG islands, shores, shelves or open sea) and promoter location status (promoter or not in promoter). The HME table was described by variation partitioning [7] for age, age acceleration and GBM classification. The results are illustrated in a Venn diagram containing the variation fractions for the three supplementary variables.

### Expression

Gene expression from RNA sequencing (RNA-seq) data (Level 3), from the TCGA GBM dataset selected for this study, was quantified for the transcript models using RSEM [29] and normalized within samples to a fixed upper quartile for TCGA. Further details are available at the DCC data portal of TCGA. Gene-level data were restricted to genes expressed in at least 70% of samples. The complete dataset was normalized by the VOOM procedure [28].

### Pathway analysis

Pathway analysis was performed by gene set enrichment analysis (GSEA) using the Molecular Signatures Database (MSigDB, v7.4.1, updated May 2021, all 8 collections) [52] using hypergeometric tests (R packages msigdbr and ClusterProfiler). Gene-sets with Bonferroni adjusted P-values ≤ 0.1 were considered significant.

### Statistical analysis

#### Detection of DMP

The associations of CpG-probes (CpGs) with age or age acceleration (Accel) were investigated by comparison of the model including the variable of interest (e.g. CpG ~ age or CpG ~ Accel) and the null model (e.g. CpG ~ 1) based on linear models with F-test. The Bonferroni procedure was used to account for multiple testing comparisons. A differentially methylated position (DMP) was defined as a candidate for which the q-value was less than 0.1.

#### Detection of functional methylation

The correlation of methylation with the expression level for each CpG-probe located in a gene promoter within 1500 nucleotides up- or down-stream of the transcription start site (TSS) was estimated by Spearman correlation test. A methylated position was defined as functional, when the q-value was less than 0.05 and the correlation coefficient was inferior to − 0.3 (negative effect on gene expression). The gene locations were based on the Homo sapiens (human) genome assembly **GRCh37** (**hg19**) available at the Genome Browser of the University of California, Santa Cruz (UCSC build hg19, http://genome.ucsc.edu) [25].

#### Cox regression model and other tests

For the continuous variables Wilcoxon test (t) or Kruskal and Wallis test (a) were used to test the differences between two or more groups. The independence between qualitative variables and groups was tested with Pearson's Chi-squared with Yates' continuity correction. Survival univariate and multivariate models were computed by Cox proportional hazards regression model [54]. The association tests with GBM classification, study origins and the interaction between these both variables were performed by model comparison using Wald’s test with sandwich estimation of the covariance matrix and F-statistic. The covariance of the models is estimated by sandwich methods with the type version *HC3* [64] to compensate heteroscedasticity. Principal component analyses (PCA) and the permutation multivariate analyses of variance (ADONIS) [1] using Euclidean distances were used to investigate the association between additional variables (e.g. age, age acceleration or GBM classification) and DNA methylation data. Analyses and graphical representations were performed using **R-4.1.2** and the R package rms and survival [17, 54].

## Results

### Patient characteristics

Age-dependent DNA methylation features of GBM were investigated in samples from patients enrolled in clinical trials for newly diagnosed GBM. They comprised samples from the Nordic trial (n = 116), treating elderly patients, and the EORTC 26,981/NCIC CE/3 and the Lausanne Pilot (LN-Pilot) trials (n = 219). The DNA methylome was established on the 450 k platform. The baseline description of the full cohorts is presented in Additional file 1: Table S1. The DNA methylation-based tumor classification [9] and its association with age is visualized in Additional file 1: Fig. S1. Of note, the patient cohort constituted of the EORTC/NCIC & LN-Pilot trials exhibited more diversity in glioma subtypes than the Nordic cohort. Detailed annotation of all samples is provided in Additional file 2.

Two additional external GBM datasets were included in the analyses. The age ranges of the patients in the four full GBM datasets, after removing all IDHmut /G-CIMP + samples, were as follows: Nordic (n = 115) 60 to 83 years (median 70, SD 4.8 years), EORTC/NCIC & LN-Pilot (n = 195) 26 to 70 years (median 55, SD 9.39 years), TCGA (n = 113) 21 to 85 years (median 62, SD 11.9 years), and DKFZ (n = 235) 18 to 86 years (median 59, SD 13.3 years). The tumors of the four datasets were classified according to the Heidelberg DNA methylation-based classifier [9]. Most GBM were classified as MES, RTK I or RTK II (88% for TCGA, 91% for the other 3 datasets), with few samples belonging to GBM-MID or GBM-G34 (no GBM-G34 in Nordic, Additional file 1: Fig. S1) or others that are now considered distinct tumor entities in the updated WHO classification 2021, and few non-GBM classifications. This study was subsequently restricted to IDHwt GBM subtypes MES, and RTK I or II, for which the baseline characteristics of the patients are summarized in Table 1 for each dataset.

**Table 1:** Baseline description and parameters of GBM datasets

Datasets	DKFZ (%)	EORTC/NCIC & LN-Pilot (%)	Nordic (%)	TCGA (%)	P-Value
N	214 (100)	177 (100)	105 (100)	99 (100)	
*Sex*					0.1994
Female	101 (47)	66 (37)	40 (38)	42 (42)	
Male	113 (53)	111 (63)	65 (62)	57 (58)	
*Patient age [years]*					< 0.0001
Min	29	27	60	33	
Max	86	70	83	85	
Mean, SD	60.34 ± 11.25	54.35 ± 8.98	70.82 ± 4.66	61.95 ± 10.64	
*DNAm age [years]*					0.0001
Min	40.02	39.56	57.90	30.11	
Max	174.01	167.42	180.10	186.71	
Mean, SD	94.65 ± 22.58	94.13 ± 26.13	107.75 ± 25.17	98.71 ± 27.72	
*Age Acc [years]*					0.1737
Min	− 13.45	− 9.50	− 7.19	− 14.89	
Max	135.01	104.14	108.49	120.71	
Mean (SD)	34.31 ± 21.25	39.78 ± 24.71	36.93 ± 25.09	36.76 ± 26.67	
*GBM subgroup*					0.0141
GBM_MES	48 (22)	66 (37)	43 (41)	31 (31)	
GBM_RTK_I	51 (24)	30 (17)	20 (19)	19 (19)	
GBM_RTK_II	115 (54)	81 (46)	42 (40)	49 (49)	
*MGMT-STP27*					0.4871
U	100 (47)	91 (51)	55 (52)	55 (56)	
M	114 (53)	86 (49)	50 (48)	44 (44)	
*MGMT score*					0.3412
Min	− 5.58	− 10.48	− 4.09	− 11.68	
Max	6.98	7.35	8.16	6.34	
Mean, SD	0.62 ± 3.59	0.22 ± 3.38	0.32 ± 3.31	− 0.23 ± 3.85	
*HM purity*					0.6177
Min	0.31	0.42	0.42	0.39	
Max	0.99	0.95	0.98	0.98	
Mean, SD	0.73 ± 0.14	0.74 ± 0.14	0.75 ± 0.14	0.75 ± 0.14	
*Global HM entropy*					0.9938
Min	0.45	0.47	0.47	0.49	
Max	0.64	0.64	0.62	0.63	
Mean, SD	0.55 ± 0.03	0.55 ± 0.03	0.55 ± 0.03	0.55 ± 0.03	
*HME Prom CpG:*					0.2526
Min	0.27	0.29	0.29	0.28	
Max	0.48	0.47	0.45	0.41	
Mean, SD	0.36 ± 0.03	0.36 ± 0.03	0.36 ± 0.03	0.36 ± 0.03	

Description of datasets used in the analyses that were restricted to IDHwt GBM WHO grade 4, 2021, and corresponding parameters determined in this study. Significant differences between the datasets were observed for patient age and DNA methylation age of GBM (DNAm age), and GBM subtypes.

Age Acc, DNAm age acceleration; GBM subgroup, methylation-based classification into mesenchymal (MES), RTK I and RTK II; Global HM entropy, human methylation based entropy of all genomic regions; HME Prom CpG, human methylation based entropy in promoter CpG islands; HM purity, human methylation based determination of sample purity; *MGMT* promoter methylation status, unmethylated U, methylated M, classified by MGMT-STP27 procedure; MGMT score, calculated with MGMT-STP27; SD, standard deviation.

The distribution of the tumors in the three GBM subclasses was significantly different between the four datasets (*p* = 0.014, chi-squared). There were less MES GBM comprised in the DKFZ dataset as compared to the others (22% less), likely due differences in patient selection of the study. There were no differences in the proportion of female and male patients, or the frequency of *MGMT* promoter methylation between the datasets.

**Fig. 1:**
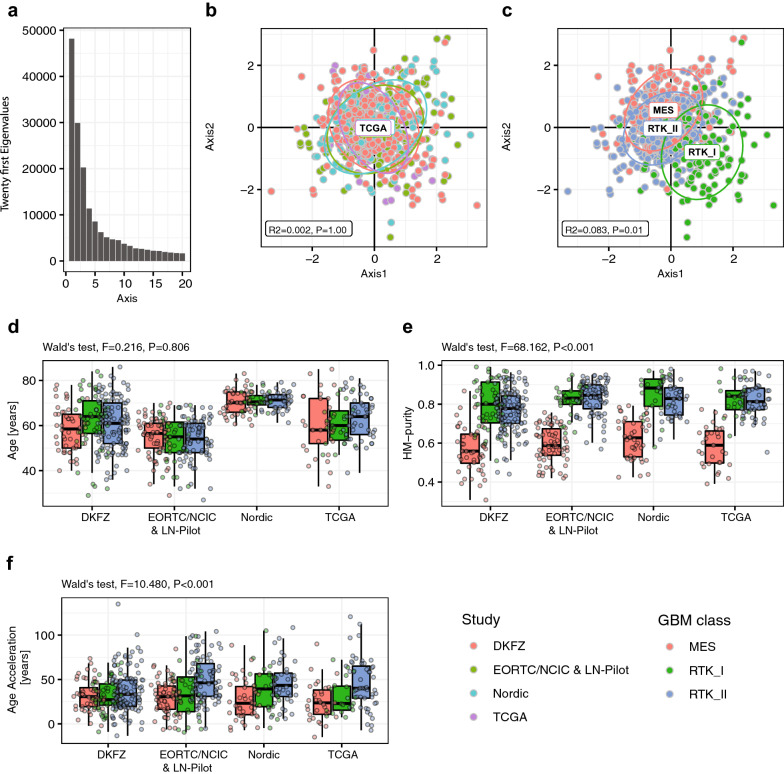
Organization of DNA methylation by dataset and GBM subtype. The representation of the twenty first eigenvalues shows the organization of the inertia structure **a** given by principal component analysis (PCA) of the DNA methylation data. The first vectorial plane, defined by the two first eigenvalues, represents 21% of the table inertia. The patient samples are represented on the first vectorial plane of the PCA (axes 1 and 2) of DNA methylation, annotated by study origin (DKFZ, red; EORTC & LN-Pilot, green; Nordic, blue; TCGA, pink; due to the overlap of the datasets, only one label is visible) **b** and methylation-based GBM classification **c**, MES, red; RTK I, green; and RTK II, blue. The R-squared (R^2^) and p-value (99 permutations, ADONIS) testing the effect between the DNA methylation data and dataset **b** and GBM subtype **c** are indicated. The boxplot representations for the patients age **d**, sample purity **e**, and DNAm age acceleration **f** are drawn, stratified by methylation-based GBM classification and study origin. No age related association with the three GBM subtypes was observed (*p* = 0.806, Wald’s test) **d**, while significant differences were found for HM-purity (*p* < 0.001, Wald’s test) **e**, and for DNAm age acceleration (*p* < 0.001, Wald’s test) **f**

After initial filtering, 361,745 CpGs were retained for subsequent analyses. After batch correction no effect of the four datasets was observed on the DNA methylation based organization of the samples as illustrated in a PCA (R^2^ = 0.002, *p* = 1.00, Fig. 1a-b). In contrast, the global organization of DNA methylation was significantly affected by GBM methylation subclasses (R^2^ = 0.083, *p* = 0.01, Fig. 1c). Similarly, no differences were observed for sample purity between the data sets (Table 1), but between the GBM subclasses (Wald’s test, *p* < 0.001, Table 2; Fig. 1e).

**Table 2:** Wald tests for models with GBM classification and study origin, and interaction

Characteristic	MES	RTK I	RTK II	GBM classification		Study		GBM Class x Study	
	Mean (sd)	Mean (sd)	Mean (sd)	*F-value	Pr(> F)	*F-value	Pr(> F)	*F-value	Pr(> F)
Patient age [yrs]	60.175 (10.853)	61.075 (11.927)	60.834 (10.83)	0.216	0.806	52.158	< 0.001	0.747	0.612
DNAm age [yrs]	89.246 (21.746)	94.538 (24.999)	104.106 (26.163)	10.717	< 0.001	4.670	0.003	1.896	0.079
Age accel [yrs]	29.072 (19.566)	33.463 (21.956)	43.273 (25.652)	10.480	< 0.001	1.457	0.225	2.456	0.024
HM-purity	0.592 (0.104)	0.817 (0.105)	0.802 (0.099)	68.162	< 0.001	2.288	0.078	1.086	0.369
Global HME	0.556 (0.029)	0.571 (0.027)	0.545 (0.029)	18.557	< 0.001	0.029	0.994	0.394	0.883
HME prom CpG	0.362 (0.032)	0.361 (0.03)	0.363 (0.029)	0.086	0.917	0.147	0.932	1.815	0.094

^*^F-values are computed with covariance matrix, estimated by sandwich estimation (type HC3) to reduce the effect of the variance heterogeneity

The lowest purity was associated with the MES GBM subtype (Fig. 1e), in line with a more pronounced fraction of tumor infiltrating cells that has been associated with this subtype [57]. The Nordic trial, recruiting only elderly patients (Table 1), introduced a significant difference of age among the four datasets (Wald’s Test, *p* < 0.001, Table 2). However, no age related association with the three GBM subtypes was observed (Wald’s Test, *p* = 0.805, Table 2, Fig. 1d). Finally, no association was observed between the GBM subtype and the WHO performance score at study entry (PS, scale 0 to 4, with higher values indicating greater disability [58]) (Cochran-Mantel-Henszel chi-squared test with stratification by study origin, *p* = 0.720). These data was available for the patients treated in the EORTC 26,981/NCIC CE/3, the LN-Pilot study and the Nordic trial, respectively (Additional file 1: Table S1).

### Age related differential methylation (DMP)

First, we analyzed the DNA methylation data for associations with the patients’ age. Age dependent methylation identified 19 CpGs (p-value ≤ 0.1, after Bonferroni correction; Additional file 1: Table S2). Of these, *ELOVL Fatty Acid Elongase 2* (*ELOVL2)* methylation at cg16867657 has previously been published as a biomarker for chronologic age (r = 0.92) [14] and is part of forensic age predictors [38, 63]. Similarly, methylation levels of *Tripartite Motif Containing 59 (TRIM59)* have been associated with chronologic age [34, 63]. Several of the probes were associated with cancer relevant genes. Three CpGs met our criteria of *functional* methylation that we defined as negative correlation of methylation with expression (≤ − 0.3 and p-value adjusted for multiple testing ≤ 0.1) of the corresponding, annotated gene. These comprised functional CpG probes for *TRIM59*, *Twist Family BHLH Transcription Factor 1 (TWIST1),* and *Nuclear Receptor Interacting Protein 3 (NRIP3)*, respectively (Additional file 1: Table S2). Functional methylation information was derived from the TCGA dataset that comprises RNA-sequencing data.

### DNA methylation age acceleration

**Fig. 2:**
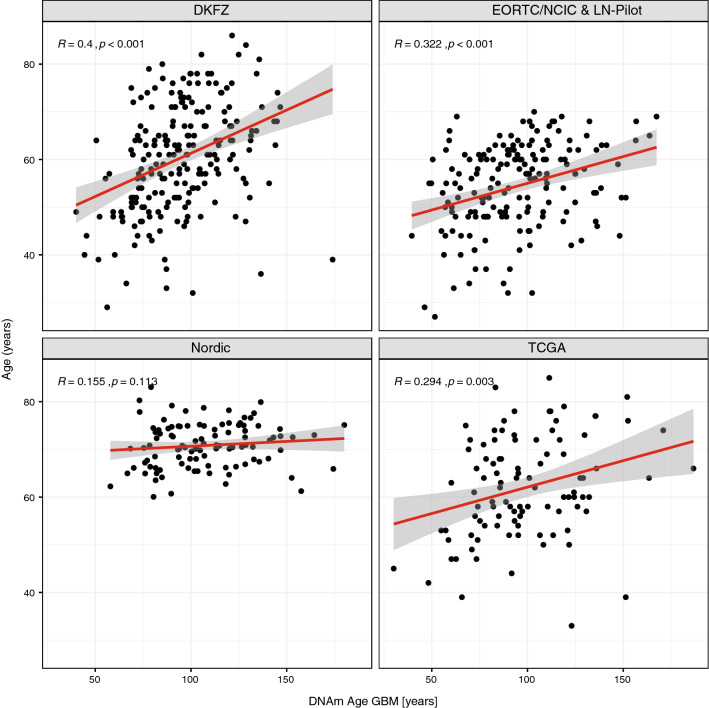
Chronological age of patients versus DNAm age of GBM. The chronological age of the patients (observed age) versus DNAm age predicted by DNA methylation data of the tumors is shown for the four datasets. The accuracy of the models is given by the Spearman’s coefficient correlation and the regression model between the observed age of the patients and the predicted age (DNAm age) of the tumors. The correlation values (|r|≤ 0.4) show strong deviation between chronological age versus DNAm age of the GBM in the four studies

Next, we determined DNAm age acceleration of the tumors that is defined as the DNAm age of the tumor, minus the patient’s age. The association of the patients’ chronologic age and the tumor DNAm age, determined with the Horvath clock, was modest, as illustrated in scatterplots for the datasets, EORTC/NCIC & LN-Pilot (r = 0.322), TCGA (r = 0.294) and the DKFZ dataset (r = 0.400), and weak for the Nordic dataset (r = 0.155) (Fig. 2). The small age range of this older population, may explain the latter (Table 1).

The age acceleration was significantly different to zero (t test, *p* < 0.001) with an averaged acceleration of 36.81 years and a standard deviation of 23.99 years. No differences in DNAm age acceleration were observed between male and female patients (*p* = 0.774, ANOVA, stratified by study). It is of note that the association between the numerous copy number variations (CNVs), characteristic for GBM, and the interrogated clock probes and DNAm age was weak. Only 7% of the variation of the DNAm age (*p* < 0.001) was explained, based on the regression of the four first axes from PCA of the clock probes, hence, excluding a strong confounding influence by CNVs.

**Fig. 3:**
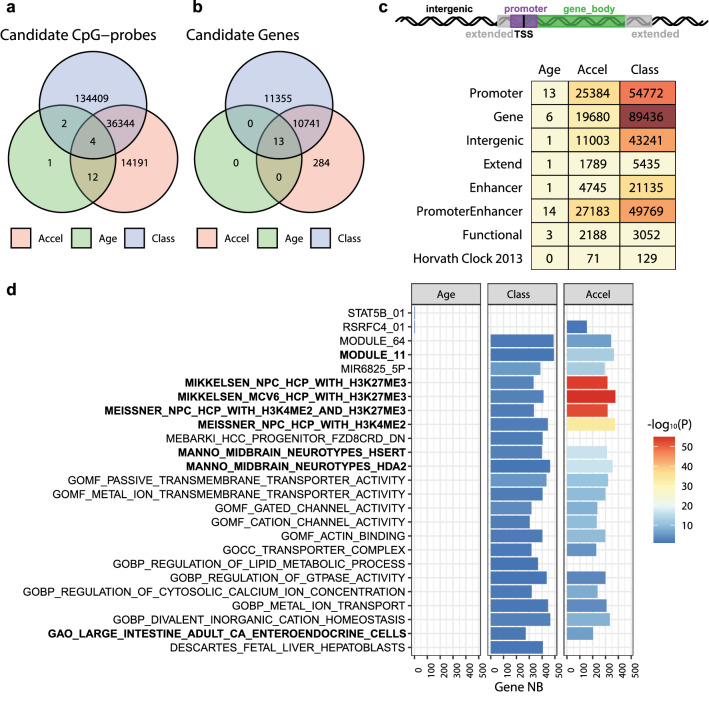
Associations of DNA methylome with age, DNAm age acceleration, and GBM subclassification. Methylome-wide association studies of the samples were performed for age (green), DNAm age acceleration (pink) and GBM classification (blue) and visualized in a Venn-diagram, for the interaction between the three sets of candidate CpG-probes **a** and the associated genes **b**. The genomic location of the selected CpGs, their association with age, DNAm age acceleration, GBM classification, the CpG functionality, and association with the Horvath clock are summarized in **c**. Gene set enrichment analysis (GSEA), using the MSigDB database, was established for candidate CpG-probes related to age, DNAm age acceleration and GBM classification. The list of gene sets **d** corresponds to the pathways significantly enriched for age and GBM classification. The number (NB) of genes per gene set is indicated and the p-value is represented by the color code. The gene lists overlapping with those associated with functional methylation (Additional file 1: Fig. S3) are highlighted in bold

A large number of CpGs associated with age acceleration were identified (DMP age accel, n = 50,551). Most of the age related CpGs (16 of 19) were also associated with age acceleration. Although by construction, DNAm age acceleration is not associated with the patients’ age (DNAm age acceleration = chronologic age of the patient minus DNAm predicted age of the tumor, see methods section). Interestingly, over 70% age acceleration associated CpGs (n = 36,348) overlapped with those associated with tumor classification (n = 170,759) into the three methylation-based GBM subtypes (MES, RTK I, RTK II) (Fig. 3a). Hence, it was not surprising that age acceleration was significantly associated with the GBM subtype (Wald’s test, *p* < 0.001, Table 2; Fig. 1f). The tumors classified as GBM RTK II, trended to exhibit higher age acceleration than the two other GBM methylation subclasses. However, the sample purity constituted a weak confounding factor, with a Spearman correlation coefficient between DNAm age acceleration and purity of r = 0.290 (*p* < 0.001), illustrated in Additional file 1: Fig. S2.

The overlap of the CpGs fulfilling all three criteria, age, DNAm age acceleration, and methylation-based subclassification, comprised 4 CpGs that also included the probe in the *ELOVL2* promoter (Additional file 1: Table S2)*.* This suggests that DNA methylation features of DNAm age acceleration are an integrative part of methylation-based tumor classification, while age seems only slightly reflected in the tumor DNA methylome. The breakdown of the selected CpGs by genome regions and function (e.g. promoter, gene body, enhancer, etc.); the three variables, age, DNAm age acceleration and classification, and affiliation with the Horvath clock, is detailed in Fig. 3c. It is of note that the observed significant association of 129 clock probes with the GBM classification was not dependent on their location on the 22 autosomes (Fisher’s test with p-value estimated by Monté-Carlo simulation, *p* = 0.558).

### Pathway analysis of DNAm age acceleration and tumor classification

Next, we were interested in the pathways to which the genes belonged that were associated with DNAm age acceleration or classification. Performing signature analysis using MiSigDB (molecular signatures database; gene set enrichment analysis [GSEA], adjusted p ≤ 0.1) and the CpGs associated with DNAm age acceleration yielded 1220 pathways. The top pathways were dominated by gene-sets characterizing epigenetic properties of neural precursor cells, or gene sets for midbrain neurotypes, and other progenitor cells, and axon development (Fig. 3d) invoking developmental features and cell of origin. The tumor classification associated CpGs yielded 23 pathways, of which most (n = 20) overlapped with those from DNAm age acceleration, in line with the large overlap of CpGs between the two criteria.

The common pathways were also dominated by gene sets characterizing epigenetic features of neural progenitors, included sets for transmembrane transporters and channels, and cancer gene sets (Module 11 and 64) (Fig. 3d). The few CpGs associated with age were linked with gene sets of downstream targets of STAT5B (Signal Transducer And Activator Of Transcription 5) and RSRFC4 (alias of MEF2A, Myocyte-Specific Enhancer Factor 2A), with 3 of the 4 genes overlapping in the two sets, the latter was also associated with DNAm age acceleration.

Subsequently we looked only at the functional CpGs (Fig. 3c), as they may shed light on associated biological mechanisms, and determined the associated pathways (GSEA, adjusted p ≤ 0.1). The “functional” pathways associated with DNAm age accel (n = 167) mostly (119, 71%) overlapped with those associated with classification (n = 294) (Additional file 1: Suppl Fig. S2). The overlapping “functional” pathways were dominated by gene-signatures characterizing epigenetic properties of neural precursor cells, or gene sets for midbrain neurotypes, and other progenitor cells [27, 35, 36]. These comprised gene sets associated with specific chromatin marks (histone H3 dimethylation mark at K4, H3K4me2, open chromatin, and trimethylation mark at K27, H3K27me3, repressive mark) associated with neural progenitor cells (NPC) [35, 36], and distinct gene signatures derived from single cell sequencing of human embryonic midbrain cells [27]. In addition, signatures of immune cells, and some cancer related signatures were comprised, the latter including the Verhaak expression signature for mesenchymal GBM (Additional file 1: Fig. S2). Interestingly, but not surprising, among the top 30 functional pathways associated with the methylation-based classification comprised three signatures of the expression based GBM classifier defined by Verhaak [56] (signatures for mesenchymal, classical, and proneural GBM). These observations support the overall consistency and biological relevance of the findings.

### Human methylation entropy (HME) and age

**Fig. 4:**
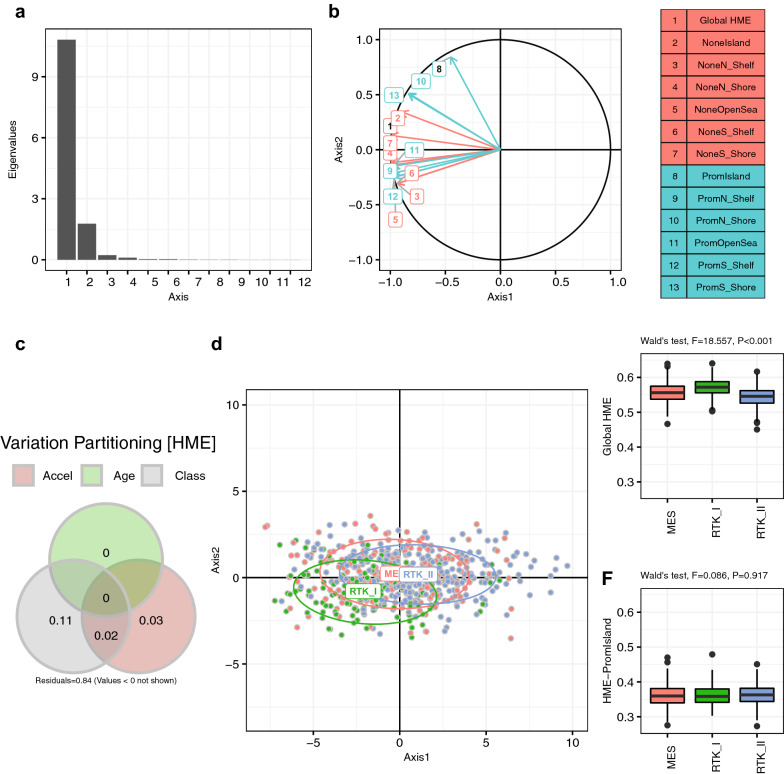
DNA methylation entropy (HME) of GBM in function of genome location. The entropy metrics based on DNA methylation (HME) was determined by genome location, constituted of the Island regions (CpG islands, shores, shelves or open sea) and promoter location (promoter or not in promoter). The Eigenvalues of the PCA of the dataset containing the HME metrics suggest that the first two dimensions explain most of the variability, **a**. The entropy (HME metrics) stratified by genome location was projected on the first vectorial plane (axes 1 & 2) of the PCA **b**. The colors of the arrows and labels indicate the genome location in (blue) or outside (red) of a promoter. **c** The contribution of DNAm age acceleration (Accel), age (Age) and GBM classification (Class) to HME was determined by variation partitioning and visualized in a Venn diagram containing the variation fractions. **d** The GBM samples are represented on the first vectorial plane of the PCA for global HME, annotated for GBM classification (MES, red; RTK I, green; RTK II, blue). Boxplot representation of global entropy **e** and entropy of CpGs located in promoter islands **f** is visualized by GBM classification. The association of classification with entropy by genome location was examined by Wald test (Additional file 1: Table S4)

Subsequently, we investigated the genome-wide variation of DNA methylation that may affect regulatory functions and genomic/epigenomic stability of the tumors. For this purpose, we used the measure of the Human Methylation Entropy (HME) that quantifies the methylation complexity for a given CpG or a given genomic region [16]. Low heterogeneity/high similarity corresponds to low entropy scores (range 0 to 1; e.g. 100% methylation, 0 entropy; 50% methylation, organized in fully methylated and fully unmethylated alleles, 0.25 entropy; 50% methylation, organized randomly, 0.65 entropy, for more details see [45]). The entropy at 12 distinct genomic regions (promoter, shore, etc.) tested, showed characteristic features by region, with strikingly lower entropy levels within CpG promoter islands (Fig. 4). This may suggest that CpG promoter islands present more homogenous DNA methylation patterns, e.g. methylated or unmethylated states, likely due to their direct regulatory function in gene expression. HME at different genomic regions (Additional file 1: Table S3) and global HME were associated with GBM subtype (Wald’s Test, *p* < 0.001, Table 2, Fig. 4e). Indeed, HME was highest in RTK I for most genomic regions. In contrast, no difference between tumor subtypes was detected for HME in the promoter CpGs Islands (Wald’s Test, *p* = 0.917, Table 2, Fig. 4f). No differences were observed between datasets at the distinct regions and over all regions combined. Finally, we performed variation partitioning of HM-entropy metrics to evaluate the contribution of age, age acceleration and GBM classification. This revealed some weak association of HM-entropy with GBM classification, explaining 13% (R^2^ = 0.131) of the variance, while age had very little impact (R^2^ = 0.005), and the contribution of DNAm age acceleration was also small (R^2^ = 0.050).

### DNA damage response (DDR) and DNAm age acceleration

Since all GBM patients, including the elderly, are treated with genotoxic therapy we had a closer look at the involvement of DNA damage response (DDR) genes in DNAm age acceleration. Evaluating only CpG probes associated with the promoter region of DDR genes as input (list as defined by Pearl et al. [41], 3947 CpGs, associated with 403 genes that might reveal age dependent treatment responses, did not yield any age related candidate CpGs.

**Fig. 5:**
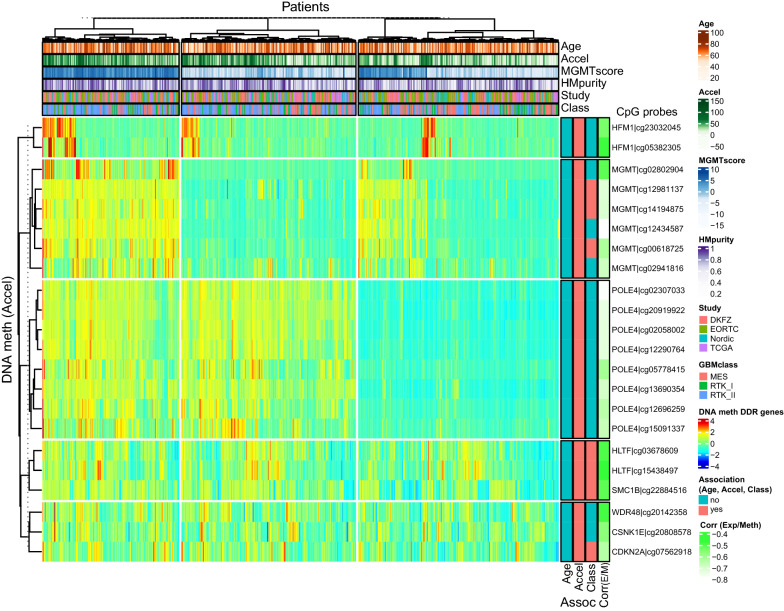
Heatmap of DNA methylation of functional CpGs in DDR genes significantly associated with DNAm age acceleration. The clustering was obtained by Ward’s algorithm using Euclidean distance based on centered and scaled data. The samples and the methylation of the functional CpGs associated with DDR genes were classified into 3 clusters (consensus k‑means clustering for 200 repetitions) and 5 clusters (consensus k‑means clustering for 200 repetitions), respectively. The samples are annotated with the patients’ age (age in years), DNAm age acceleration (Accel, in years), *MGMT* methylation score (MGMTscore), Purity index based on DNA methylation (HMpurity), study origin (study) and GBM classification (Class). The probes are annotated for statistical significance associated with age (Age), DNAm age acceleration (Accel) and GBM classification (Class); yes, red; no, blue. The Spearman’s correlation of methylation with expression (measure of functionality) is given for each CpG, Corr (E/M). The clustering is dominated by the high correlation of DNA methylation among the functional CpGs of individual genes

DMPs related to DNAm age acceleration comprised 206 CpGs in 109 DDR genes whereof 22 CpGs in 8 genes were functional and comprised among others, the gene encoding an accessory subunit of DNA Polymerase Epsilon (*POLE4)*, the gene encoding Cyclin Dependent Kinase Inhibitor 2A *(CDKN2A), MGMT,* and the gene *Structural Maintenance of Chromosomes 1B (SMC1B)* visualized in a heatmap (Fig. 5) (Additional file 1: Table S4). DMPs associated with tumor classification consisted of 272 CpGs in 138 DDR genes whereof 22 in 12 genes were functional (Additional file 1: Table S5). Four genes with functional CpGs were associated with both DNAm age acceleration and tumor classification and comprised *CDKN2A*, *MGMT*, the gene for the Helicase like Transcription Factor (*HTLF*), and *SMC1B,* as annotated in Fig. 5. *CDKN2A* is a prominent tumor suppressor gene, frequently affected by homozygous deletions (HD) in IDHmut GBM [8], Therefore we tested the methylation of the CpG-probe cg07562918 (M-value) located in the *CDKN2A* gene that was significantly associated with age acceleration and tumor classification, for association with the HD-status. Methylation of this CpG was significantly associated with HD of the *CDKN2A* gene (Wilcoxon test *p* < 0.001). However, no significant direct effect of the HD status of the *CDKN2A* gene was identified on DNAm age acceleration (Wilcoxon test, *p* = 0.163; Additional file 1: Fig. S4).

### Age acceleration and outcome

Finally, we integrated DNAm age acceleration into the multivariable model for outcome for the two clinical trial cohorts, and included the following parameters: treatment (TMZ/RT→TMZ for EORTC/NCIC & LN-Pilot; TMZ for Nordic), MGMT status (*MGMT* methylation), and the interaction between treatment and *MGMT* methylation (predictive factor). DNAm age acceleration was significant in both cohorts, EORTC/NCIC & LN-Pilot *p* = 0.0138 and Nordic *p* = 0.00161 (Table 3). The predictive value of *MGMT* methylation (interaction between treatment and *MGMT* methylation) was confirmed in the EORTC/NCIC & LN-Pilot cohort (*p* < 0.0001). The interaction term in the Nordic cohort was not significant (*p* = 0.09). However, the number of *MGMT* methylated patients in the TMZ-arm was very small (n = 13), suggesting lack of power for this test.

**Table 3:** Multivariable Cox regression models including age acceleration, GBM classification, global HM-entropy (HME) and HME at promoter CpGs

Model	Variables	EORTC/NCIC & LN-Pilot (N = 177)				Nordic study (N = 105)			
		Modality	HR	z-value	Pr( >|z|)	Modality	HR	z-value	Pr( >|z|)
*Age acceleration [years]*									
	Treatment (TRT)	TMZ + RT	1.34862	1.31435	0.18873	TMZ	1.06845	0.23966	0.81059
	MGMT	MGMTmeth	0.77273	− 1.10615	0.26866	MGMTmeth	1.13894	0.50912	0.61067
	Age Acceleration		0.99124	− 2.46259	**0.01379**		0.98549	− 3.15493	**0.00161**
	TRT x MGMT	TMZ + RT x MGMTmeth	0.27215	− 3.94628	**0.00008**	TMZ x MGMTmeth	0.47267	− 1.66716	0.09548
*GBM classification*									
	Treatment (TRT)	TMZ + RT	1.19859	0.80597	0.42026	TMZ	1.12226	0.41692	0.67674
	MGMT	MGMTmeth	0.70503	− 1.52048	0.12839	MGMTmeth	1.05817	0.22379	0.82292
	GBM classification	GBM_RTK_I	1.54086	1.91764	0.05516	GBM_RTK_I	1.12308	0.41675	0.67686
		GBM_RTK_II	0.91674	− 0.48386	0.62848	GBM_RTK_II	0.52912	− 2.63579	**0.00839**
	TRT x MGMT	TMZ + RT x MGMTmeth	0.31994	− 3.55697	**0.00038**	TMZ x MGMTmeth	0.53507	− 1.39736	0.16230
*Global HME*									
	Treatment (TRT)	TMZ + RT	1.12690	0.55400	0.57958	TMZ	1.08996	0.30807	0.75803
	MGMT	MGMTmeth	0.69179	− 1.61442	0.10644	MGMTmeth	0.89983	− 0.42203	0.67300
	HME		3.18126	0.49623	0.61973		164.80788	1.64897	0.09915
		TMZ + RT x MGMTmeth	0.34584	− 3.38245	**0.00072**	TMZ x MGMTmeth	0.54313	− 1.36378	0.17264
*HME promoter CpG*									
	Treatment (TRT)	TMZ + RT	1.15106	0.65024	0.51554	TMZ	1.17499	0.58063	0.56149
	MGMT	Methylated	0.73235	− 1.33193	0.18288	MGMTmeth	0.95937	− 0.16478	0.86912
	HME prom CpG		0.04405	− 1.14097	0.25388		0.16488	− 0.58312	0.55982
	TRT x MGMT	TMZ + RT x MGMTmeth	0.33173	− 3.48833	**0.00049**	TMZ x MGMTmeth	0.53779	− 1.39207	0.16390

Cox models, adjusted for Treatment (TRT), methylation status of *MGMT* promoter (MGMT), and the interaction of these two variables (TRT X MGMT)

HME, human methylation entropy; HR, hazard ratio; MGMTmeth, methylated *MGMT;* Pr(( >|z|), p-value for z-statistics; P-value <0.5, bold

## Discussion

In this study, we investigated the DNA methylome of IDHwt GBM for age related differences that could hint at predictive or prognostic factors or indicate particular vulnerabilities for treatments in elderly patients. However, we found no strong direct associations, although we identified the methylation probe in *ELOVL2* that has reportedly the highest association with age as a single marker [5, 14] and *TRIM59* methylation, another robust marker for aging [34, 63]. *ELOVL2* is a GBM-relevant gene [15, 44]. However, methylation of this probe was not functional according to our analysis. *TRIM59* was among the genes associated with functional methylation. It encodes a protein with ubiquitin-transferase activity, and it has been associated with various regulatory processes and maybe involved in innate immune regulation.

We then used the metric of DNAm age and age acceleration proposed by the Horvath DNA methylation clock that has been trained on multiple tissues using the HM-27 k array (does not comprise CpG probes *associated/annotated* with *ELOVL2*), and has been reported to be highly accurate to predict chronologic age (r = 0.92) [14]. This methylation clock is considered independent of tissue type and mitotic potential [21, 23]. Various epigenetic clocks have been developed with potential as biomarkers. The aim is not only to determine accurate chronologic age, but to develop biological clocks for specific purposes, such as tissue specific sensors of disease, risk of disease, including cancer risk, or external stress, such as smoking history, or all-cause mortality (GrimAge) (reviewed in [5, 12, 23, 34]).

DNAm age acceleration observed for GBM averaged at almost 40 years, with a wide variability. No association was found with the patients’ sex.

The acceleration associated gene sets, identified using the MiSigDB, comprised several distinct cell type-specific signatures obtained by single cell sequencing of cells derived from embryonic ventral midbrain [27]. Other signatures reflected epigenetic features at developmentally regulated genes with high CpG density promoters that are characterized by bivalent histone marks (both active H3K4me3 and repressive H3K27me3 marks) that are poised but silent in ES (embryonic stem) cells and active in NPC, such as the genes encoding the Oligodendrocyte Transcription Factor 1 and 2 (*Olig1* and *Olig 2)*. Changes in the pattern of histone marks have a strong influence on DNA methylation. The signatures identified here, associated with DNAm age acceleration reflected a change, with loss of the active mark H3K4me3 and retention of H3K27me2 and/or the repressive mark H3K27me3 that has been associated with partial increase of DNA methylation [35]. Hypermethylation of such regulatory genes that are active under differentiation are found preferentially aberrantly methylated and thereby silenced in cancer. Such genes are reported to be regulated by thritorax-group and/or polycomb-repressive complex 2 (PRC2) proteins. Furthermore, CpGs whose methylation increases with age at specific locations, have been found overrepresented near polycomb target genes and bivalent chromatin domains. These genes are of importance for stemness and cell differentiation and have been found frequently methylated in cancer [21, 53]. Beside these insights into epigenetic mechanisms associated with age acceleration and the neurotypes specific signatures [27, 35, 36], suggestive of developmental features and cell of origin, the analyses also yielded signatures for immune cells, cell death, and cancer. The biological relevance is further supported by the fact that a subset of these signatures overlapped with those associated with functional methylation, implicating that the observed increased methylation was negatively associated with expression of the corresponding genes, affecting related pathways (Fig. 3d; Additional file 1: Fig. S2). The investigation of mechanisms and biological consequences reflected in methylation aging clocks is an active field of research that has been extensively reviewed elsewhere [5, 12, 47].

When associating the observed DNAm age acceleration of the GBM with patient outcome, we observed a significant effect, when analyzing the cohorts treated in clinical trials. Increased age acceleration in these IDHwt GBM was associated with better outcome, raising the question of the biological meaning of *DNAm age acceleration* in the context of these tumors. Therefore, we consider tumor related DNAm age acceleration as a measure for epigenetic distance associated with tumor development and GBM subtype. This is in accordance with the observation that DNAm age acceleration associated probes overlap largely with those associated with methylation–based GBM classification.

It has been suggested that deterioration of epigenetic maintenance contributes to age related changes [23], and more recently, it has been proposed that DNA break-induced epigenetic drift may contribute to aging [18]. Given the gross structural changes observed in GBM this is an interesting hypothesis. However, the numerous CNVs, characteristic of GBM, only explained a minor part (7%) of DNAm age of the tumors. Along the same lines, while a subset of clock probes was significantly associated with the methylation–based GBM classification, this was not dependent on their chromosomal location. Hence, this measure seems to comprehensively capture GBM related epigenetic changes that are associated in part with the GBM subclassification. The DNA methylation-based GBM classification is constituted of multidimensional information reflected by DNA methylation that are contributed by biological features such as purity, age acceleration, HM-entropy (variability of the DNA methylation), and other factors. The purity, reflecting infiltrating immune cells, measured by means of a DNA methylation variation, contributes to the differentiation of MES GBM samples, while DNAm age acceleration facilitates the differentiation of the RTK II GBM samples from those classified as RTK I and MES, and the HME discerns RTK I from the two others. Interestingly, among the significant pathways associated functional methylation and tumor classification, we identified previously reported expression-based GBM classification signatures [56]. This underlines the strong functional implication of DNA methylation on the expression phenotypes of the tumors and the coherence of the results.

The HM entropy metrics (12 distinct regions, and global entropy) exhibited genome-location dependent variation explained in part by the GBM subtypes (13%), but was basically not affected by age (0.5%). As an exception, HM entropy at promoter associated locations was lowest, and was not different across the GBM subtypes, indicating little permissiveness for variation in accordance with direct functional implications of methylated versus unmethylated status of promoters on gene expression.

Finally, we were interested in the DDR genes with functional methylation associated with DNAm age acceleration and classification that may yield mechanistic insights in the context of the genotoxic treatments that are part of the standard of care. Enhanced *POLE4* methylation (8 functional probes) was associated with DNAm age acceleration (Additional file 1: Figure S3). Deletion of *Pole-4* that encodes an accessory subunit of the DNA polymerase epsilon complex, has been reported to have no strong effects on its own in worms, however, in absence of the gene encoding the regulator of telomere elongation helicase 1 *(rtel-1)* apparently led to synthetic lethality due to impaired homologous recombination (HR) [6]. Similarly, six functional probes of *MGMT* were associated with DNAm age acceleration, whereof three were also associated with GBM subclassification. Functional probes in *CDKN2A, HLTF,* and *SMCB1* were also associated with both. Other functional probes were only associated with tumor classification, e.g. the gene encoding the Fanconi anemia complementation group M protein *(FANCM)* or the gene encoding the AlkB Homolog 1, Histone H2A Dioxygenase *(ALKBH1).* The former is involved in homology directed DNA repair, and the latter takes part in the repair of DNA alkylation damage and has recently been associated with the regulation of the level of N^6^-methyladenine (N^6^-mA) DNA modifications, implicated in epigenetic regulation of gene expression relevant in GBM [61]. While *MGMT* methylation is a known predictive factor for responsiveness to the alkylating agent TMZ in GBM, it remains to be explored, whether any of the other identified probes and their associated genes and pathways, indicate potentially actionable vulnerabilities.

In conclusion, our efforts to identify epigenetic differences in GBM of elderly patients revealed that once all high grade gliomas not classified as GBM IDHwt WHO grade 4 are removed, the distribution and spectrum of the GBM subtypes seems to be comparable across adult age, at least from a DNA methylation point of view (Fig. 1). DNAm differences of the tumors quantified as age acceleration or HME overlap with features of tumor classification, while age is hardly reflected. Interestingly, the epigenetic distance measured as DNAm age acceleration was associated with better outcome in the cohorts treated in clinical trials also for elderly GBM patients. However, our analyses yielded no molecular evidence for age related differences that would advocate different treatment modalities in elderly GBM patients.

## Supplementary Information

**Additional file. 1.** **Table S1.**Description of full EORTC/NCIC & LN-Pilot and Nordic datasets. **Table S2. **DNA methylation associated with patient age. **Table S3. **Associations of methylation based entropy by genomic region and GBM classification. **Table S4. **Functional CpGs located on DDR genes, associated with Age Acceleration. **Table S5. **Functional CpGs located on DDR genes, associated with GBM classification. **Fig. S1 **Methylation-based classification of tumors of all patients from EORTC/NCIC & LN-Pilot and Nordic studies. **Fig. S2 **Impact of sample purity on measures of DNAm age acceleration, and HM entropy. **Fig. S3 **Pathways associated with functional methylation related to DNAm age acceleration and GBM classification. **Fig. S4 ***CDKN2A* CNV and DNAm age acceleration.

**Additional file. 2.** Sample annotation of full EORTC/NCIC & LN-Pilot and Nordic datasets.

## Data Availability

The datasets generated and/or analyzed during the current study are available in the Gene Expression Omnibus (GEO) repository. The datasets are available at GEO (http://www.ncbi.nlm.nih.gov/geo/) under the accession numbers GSE195684 for the Nordic trial samples, and the samples from the LN-Pilot trial and the EORTC 26,981/NCIC CE.3 trial are available under GSE195640, or GSE60274. The latter comprises data from a subset of GBM samples of the EORTC-NCIC & Pilot trials and 5 non-tumoral brain tissue samples that we have previously published [26]. Methylation data from an additional 5 non-tumoral brain tissues used, is available under GSE104293 [3]. External datasets comprised the GBM dataset from The Cancer Genome Atlas for which RNA-seq and HM-450 k data are available and using corresponding annotations [8] (TCGA; n = 113; dbGaP accession number phs000178.v9.p8; http://cancergenome.nih.gov), and a GBM set with HM-450 k data from the DKFZ [9] (n = 235; GEO accession number GSE109381).

## Abbreviations

Accel: DNAm age acceleration
*ALKBH1*: AlkB Homolog 1Histone H2A Dioxygenase gene
ANOVA: Analysis of variance
*CDKN2A*: Cyclin Dependent Kinase Inhibitor 2A gene
CNV: Copy number variation
CpG: Cytosine nucleotide followed by guanine nucleotide
DDR: DNA damage response
DKFZ: Deutsches Krebsforschungszentum
DMP: Differentially methylated position
DNAm age: DNA methylation age
*ELOVL2*: *ELOVL Fatty Acid Elongase 2 gene*
EORTC: European Organisation for Research and Treatment of Cancer
ES cells: Embryonic stem cells
*FANCM*: Fanconi anemia complementation group M protein gene
FFPE: Formalin fixed paraffin embedded
GBM: Glioblastoma
G-CIMP: Glioma CpG island methylator phenotype
GEO: Gene Expression Omnibus
GSEA: Gene set enrichment analysis
H3K4me2: Histone H3 dimethylation mark at lysine (K) 4
H3K27me3: Histone H3 trimethylation mark at lysine (K) 27
*HTLF*: Helicase like Transcription Factor gene
*HM-27 k/450 k*: human methylation bead chip version 27 k / 450 k
HME: Human methylation entropy
HR: Homologous recombination
*IDH1*: Isocitrate dehydrogenase gene 1
IDHmut / IDHwt: Mutant / wild type forms of IDH1 or 2
LN-Pilot: Lausanne-Pilot trial
MES: Mesenchymal subtype
*MGMT*: O^6^-methylguanin DNA methyl transferase gene
MiSigDB: Molecular signatures database
N^6^-mA: N^6^-methyladenine
NCIC: National Cancer Institute of Canada
Noob: Normal-exponential out-of-band
NPC: Neuronal precursor cell
*NRIP3*: Nuclear Receptor Interacting Protein 3 gene
*Olig 1* and *Olig 2*: Genes encoding Oligodendrocyte Transcription Factor 1 and 2
PCA: Principle component analysis
*POLE4*: Accessory subunit of DNA Polymerase Epsilon gene
RNA-seq: RNA sequencing
RSRFC4: Alias of MEF2A (Myocyte-Specific Enhancer Factor 2A)
RT: Radio therapy
*rtel-1*: Regulator of telomere elongation helicase 1 gene (mouse)
RTK I/II: Receptor tyrosine kinase type I / II
*SMC1B*: Structural Maintenance of Chromosomes 1B gene
STAT5B: Signal Transducer And Activator Of Transcription 5
TCGA: The Cancer Genome Atlas
TMZ: Temozolomide
*TRIM59*: Tripartite Motif Containing 59 gene
TSS: Transcription start site
*TWIST1*: Twist Family BHLH Transcription Factor 1 gene
UCSC build hg19: University of California at Santa Cruz human genome build 19
VOOM: Mean–variance modelling at the observational level
WHO: World Health Organization
WHO-CNS-5: WHO classification of central nervous system tumors update 5 2021
WHO PS: WHO performance score

## References

[1] Anderson MJ (2001). A new method for non-parametric multivariate analysis of variance. Austral Ecol.

[2] Bady P, Delorenzi M, Hegi ME (2016). Sensitivity analysis of the MGMT-STP27 model and impact of genetic and epigenetic context to predict the MGMT methylation status in gliomas and other tumors. J Mol Diagn.

[3] Bady P, Kurscheid S, Delorenzi M, Gorlia T, van den Bent MJ, Hoang-Xuan K, Vauleon E, Gijtenbeek A, Enting R, Thiessen B (2018). The DNA methylome of DDR genes and benefit from RT or TMZ in IDH mutant low-grade glioma treated in EORTC 22033. Acta Neuropathol.

[4] Bady P, Sciuscio D, Diserens AC, Bloch J, van den Bent MJ, Marosi C, Dietrich PY, Weller M, Mariani L, Heppner FL (2012). MGMT methylation analysis of glioblastoma on the Infinium methylation BeadChip identifies two distinct CpG regions associated with gene silencing and outcome, yielding a prediction model for comparisons across datasets, tumor grades, and CIMP-status. Acta Neuropathol.

[5] Bell CG, Lowe R, Adams PD, Baccarelli AA, Beck S, Bell JT, Christensen BC, Gladyshev VN, Heijmans BT, Horvath S (2019). DNA methylation aging clocks: challenges and recommendations. Genome Biol.

[6] Bellelli R, Youds J, Borel V, Svendsen J, Pavicic-Kaltenbrunner V, Boulton SJ (2020). Synthetic lethality between DNA polymerase epsilon and RTEL1 in metazoan DNA replication. Cell Rep.

[7] Borcard D, Legendre P, Drapeau P (1992). Partialling out the spatial component of ecological variation. Ecology.

[8] Brennan CW, Verhaak RG, McKenna A, Campos B, Noushmehr H, Salama SR, Zheng S, Chakravarty D, Sanborn JZ, Berman SH (2013). The somatic genomic landscape of glioblastoma. Cell.

[9] Capper D, Jones DTW, Sill M, Hovestadt V, Schrimpf D, Sturm D, Koelsche C, Sahm F, Chavez L, Reuss DE (2018). DNA methylation-based classification of central nervous system tumours. Nature.

[10] Ceccarelli M, Barthel FP, Malta TM, Sabedot TS, Salama SR, Murray BA, Morozova O, Newton Y, Radenbaugh A, Pagnotta SM (2016). Molecular profiling reveals biologically discrete subsets and pathways of progression in diffuse glioma. Cell.

[11] Du P, Zhang X, Huang CC, Jafari N, Kibbe WA, Hou L, Lin SM (2010). Comparison of Beta-value and M-value methods for quantifying methylation levels by microarray analysis. BMC Bioinform.

[12] Field AE, Robertson NA, Wang T, Havas A, Ideker T, Adams PD (2018). DNA methylation clocks in aging: categories, causes, and consequences. Mol Cell.

[13] Fortin JP, Labbe A, Lemire M, Zanke BW, Hudson TJ, Fertig EJ, Greenwood CM, Hansen KD (2014). Functional normalization of 450k methylation array data improves replication in large cancer studies. Genome Biol.

[14] Garagnani P, Bacalini MG, Pirazzini C, Gori D, Giuliani C, Mari D, Di Blasio AM, Gentilini D, Vitale G, Collino S (2012). Methylation of ELOVL2 gene as a new epigenetic marker of age. Aging Cell.

[15] Gimple RC, Kidwell RL, Kim LJY, Sun T, Gromovsky AD, Wu Q, Wolf M, Lv D, Bhargava S, Jiang L (2019). Glioma stem cell-specific superenhancer promotes polyunsaturated fatty-acid synthesis to support EGFR signaling. Cancer Discov.

[16] Hannum G, Guinney J, Zhao L, Zhang L, Hughes G, Sadda S, Klotzle B, Bibikova M, Fan JB, Gao Y (2013). Genome-wide methylation profiles reveal quantitative views of human aging rates. Mol Cell.

[17] Harrell FE (2015). Regression modeling strategies.

[18] Hayano M, Yang J-H, Bonkowski MS, Amorim JA, Ross JM, Coppotelli G, Griffin P, Chew YC, Guo W, Yang X (2019). DNA break-induced epigenetic drift as a cause of mammalian aging. SSRN Electron J.

[19] Hegi ME, Diserens AC, Godard S, Dietrich PY, Regli L, Ostermann S, Otten P, Van Melle G, de Tribolet N, Stupp R (2004). Clinical trial substantiates the predictive value of O-6-methylguanine-DNA methyltransferase promoter methylation in glioblastoma patients treated with temozolomide. Clin Cancer Res.

[20] Hegi ME, Diserens AC, Gorlia T, Hamou MF, de Tribolet N, Weller M, Kros JM, Hainfellner JA, Mason W, Mariani L (2005). MGMT gene silencing and benefit from temozolomide in glioblastoma. N Engl J Med.

[21] Horvath S (2013). DNA methylation age of human tissues and cell types. Genome Biol.

[22] Horvath S (2015). Erratum to: DNA methylation age of human tissues and cell types. Genome Biol.

[23] Horvath S, Raj K (2018). DNA methylation-based biomarkers and the epigenetic clock theory of ageing. Nat Rev Genet.

[24] Johnson WE, Li C, Rabinovic A (2007). Adjusting batch effects in microarray expression data using empirical Bayes methods. Biostatistics.

[25] Kent WJ, Sugnet CW, Furey TS, Roskin KM, Pringle TH, Zahler AM, Haussler D (2002). The human genome browser at UCSC. Genome Res.

[26] Kurscheid S, Bady P, Sciuscio D, Samarzija I, Shay T, Vassallo I, Criekinge WV, Daniel RT, van den Bent MJ, Marosi C (2015). Chromosome 7 gain and DNA hypermethylation at the HOXA10 locus are associated with expression of a stem cell related HOX-signature in glioblastoma. Genome Biol.

[27] La Manno G, Gyllborg D, Codeluppi S, Nishimura K, Salto C, Zeisel A, Borm LE, Stott SRW, Toledo EM, Villaescusa JC (2016). Molecular diversity of midbrain development in mouse, human, and stem cells. Cell.

[28] Law CW, Chen Y, Shi W, Smyth GK (2014). voom: Precision weights unlock linear model analysis tools for RNA-seq read counts. Genome Biol.

[29] Li B, Dewey CN (2011). RSEM: accurate transcript quantification from RNA-Seq data with or without a reference genome. BMC Bioinformatics.

[30] Liao P, Ostrom QT, Stetson L, Barnholtz-Sloan JS (2018). Models of epigenetic age capture patterns of DNA methylation in glioma associated with molecular subtype, survival, and recurrence. Neuro Oncol.

[31] Louis DN, Perry A, Reifenberger G, von Deimling A, Figarella-Branger D, Cavenee WK, Ohgaki H, Wiestler OD, Kleihues P, Ellison DW (2016). The 2016 World Health Organization classification of tumors of the central nervous system: a summary. Acta Neuropathol.

[32] Louis DN, Perry A, Wesseling P, Brat DJ, Cree IA, Figarella-Branger D, Hawkins C, Ng HK, Pfister SM, Reifenberger G (2021). The 2021 WHO classification of tumors of the central nervous system: a summary. Neuro Oncol.

[33] Malmstrom A, Gronberg BH, Marosi C, Stupp R, Frappaz D, Schultz H, Abacioglu U, Tavelin B, Lhermitte B, Hegi ME (2012). Temozolomide versus standard 6-week radiotherapy versus hypofractionated radiotherapy in patients older than 60 years with glioblastoma: the Nordic randomised, phase 3 trial. Lancet Oncol.

[34] McCartney DL, Min JL, Richmond RC, Lu AT, Sobczyk MK, Davies G, Broer L, Guo X, Jeong A, Jung J (2021). Genome-wide association studies identify 137 genetic loci for DNA methylation biomarkers of aging. Genome Biol.

[35] Meissner A, Mikkelsen TS, Gu H, Wernig M, Hanna J, Sivachenko A, Zhang X, Bernstein BE, Nusbaum C, Jaffe DB (2008). Genome-scale DNA methylation maps of pluripotent and differentiated cells. Nature.

[36] Mikkelsen TS, Ku M, Jaffe DB, Issac B, Lieberman E, Giannoukos G, Alvarez P, Brockman W, Kim TK, Koche RP (2007). Genome-wide maps of chromatin state in pluripotent and lineage-committed cells. Nature.

[37] Moran S, Martinez-Cardus A, Sayols S, Musulen E, Balana C, Estival-Gonzalez A, Moutinho C, Heyn H, Diaz-Lagares A, de Moura MC (2016). Epigenetic profiling to classify cancer of unknown primary: a multicentre, retrospective analysis. Lancet Oncol.

[38] Naue J, Hoefsloot HCJ, Mook ORF, Rijlaarsdam-Hoekstra L, van der Zwalm MCH, Henneman P, Kloosterman AD, Verschure PJ (2017). Chronological age prediction based on DNA methylation: Massive parallel sequencing and random forest regression. Forensic Sci Int Genet.

[39] Noushmehr H, Weisenberger DJ, Diefes K, Phillips HS, Pujara K, Berman BP, Pan F, Pelloski CE, Sulman EP, Bhat KP (2010). Identification of a CpG island methylator phenotype that defines a distinct subgroup of glioma. Cancer Cell.

[40] Ostrom QT, Cioffi G, Gittleman H, Patil N, Waite K, Kruchko C, Barnholtz-Sloan JS (2019). CBTRUS statistical report: Primary brain and other central nervous system tumors diagnosed in the United States in 2012–2016. Neuro Oncol.

[41] Pearl LH, Schierz AC, Ward SE, Al-Lazikani B, Pearl FM (2015). Therapeutic opportunities within the DNA damage response. Nat Rev Cancer.

[42] Perry JR, Laperriere N, O'Callaghan CJ, Brandes AA, Menten J, Phillips C, Fay M, Nishikawa R, Cairncross JG, Roa W (2017). Short-course radiation plus temozolomide in elderly patients with glioblastoma. N Engl J Med.

[43] Roa W, Brasher PM, Bauman G, Anthes M, Bruera E, Chan A, Fisher B, Fulton D, Gulavita S, Hao C (2004). Abbreviated course of radiation therapy in older patients with glioblastoma multiforme: a prospective randomized clinical trial. J Clin Oncol.

[44] Saurty-Seerunghen MS, Bellenger L, El-Habr EA, Delaunay V, Garnier D, Chneiweiss H, Antoniewski C, Morvan-Dubois G, Junier MP (2019). Capture at the single cell level of metabolic modules distinguishing aggressive and indolent glioblastoma cells. Acta Neuropathol Commun.

[45] Scherer M, Nebel A, Franke A, Walter J, Lengauer T, Bock C, Muller F, List M (2020). Quantitative comparison of within-sample heterogeneity scores for DNA methylation data. Nucleic Acids Res.

[46] Shannon CE (1948). A mathematical theory of communication. Bell Syst Tech J.

[47] Simpson DJ, Olova NN, Chandra T (2021). Cellular reprogramming and epigenetic rejuvenation. Clin Epigenet.

[48] Stupp R, Dietrich PY, Ostermann Kraljevic S, Pica A, Maillard I, Maeder P, Meuli R, Janzer R, Pizzolato G, Miralbell R (2002). Promising survival for patients with newly diagnosed glioblastoma multiforme treated with concomitant radiation plus temozolomide followed by adjuvant temozolomide. J Clin Oncol.

[49] Stupp R, Hegi ME, Mason WP, van den Bent MJ, Taphoorn MJ, Janzer RC, Ludwin SK, Allgeier A, Fisher B, Belanger K (2009). Effects of radiotherapy with concomitant and adjuvant temozolomide versus radiotherapy alone on survival in glioblastoma in a randomised phase III study: 5-year analysis of the EORTC-NCIC trial. Lancet Oncol.

[50] Stupp R, Mason WP, van den Bent MJ, Weller M, Fisher B, Taphoorn MJB, Belanger K, Brandes AA, Cairncross JG, Marosi C (2005). Radiotherapy plus concomitant and adjuvant temozolomide for glioblastoma. N Engl J Med.

[51] Sturm D, Witt H, Hovestadt V, Khuong-Quang DA, Jones DT, Konermann C, Pfaff E, Tonjes M, Sill M, Bender S (2012). Hotspot mutations in H3F3A and IDH1 define distinct epigenetic and biological subgroups of glioblastoma. Cancer Cell.

[52] Subramanian A, Tamayo P, Mootha VK, Mukherjee S, Ebert BL, Gillette MA, Paulovich A, Pomeroy SL, Golub TR, Lander ES (2005). Gene set enrichment analysis: a knowledge-based approach for interpreting genome-wide expression profiles. Proc Natl Acad Sci U S A.

[53] Teschendorff AE, Menon U, Gentry-Maharaj A, Ramus SJ, Weisenberger DJ, Shen H, Campan M, Noushmehr H, Bell CG, Maxwell AP (2010). Age-dependent DNA methylation of genes that are suppressed in stem cells is a hallmark of cancer. Genome Res.

[54] Therneau TM, Grambsch PM (2000). Modeling survival data: extending the cox model.

[55] van de Wiel MA, Kim KI, Vosse SJ, van Wieringen WN, Wilting SM, Ylstra B (2007). CGHcall: calling aberrations for array CGH tumor profiles. Bioinformatics.

[56] Verhaak RG, Hoadley KA, Purdom E, Wang V, Qi Y, Wilkerson MD, Miller CR, Ding L, Golub T, Mesirov JP (2010). Integrated genomic analysis identifies clinically relevant subtypes of glioblastoma characterized by abnormalities in PDGFRA, IDH1, EGFR, and NF1. Cancer Cell.

[57] Wang Q, Hu B, Hu X, Kim H, Squatrito M, Scarpace L, deCarvalho AC, Lyu S, Li P, Li Y (2017). Tumor evolution of glioma-intrinsic gene expression subtypes associates with immunological changes in the microenvironment. Cancer Cell.

[58] West HJ, Jin JO (2015). JAMA oncology patient page. Performance status in patients with cancer. JAMA Oncol.

[59] Wick A, Kessler T, Elia AEH, Winkler F, Batchelor TT, Platten M, Wick W (2018). Glioblastoma in elderly patients: solid conclusions built on shifting sand?. Neuro Oncol.

[60] Wiestler B, Claus R, Hartlieb SA, Schliesser MG, Weiss EK, Hielscher T, Platten M, Dittmann LM, Meisner C, Felsberg J (2013). Malignant astrocytomas of elderly patients lack favorable molecular markers: an analysis of the NOA-08 study collective. Neuro Oncol.

[61] Xie Q, Wu TP, Gimple RC, Li Z, Prager BC, Wu Q, Yu Y, Wang P, Wang Y, Gorkin DU (2018). N(6)-methyladenine DNA modification in glioblastoma. Cell.

[62] Yang Z, Wong A, Kuh D, Paul DS, Rakyan VK, Leslie RD, Zheng SC, Widschwendter M, Beck S, Teschendorff AE (2016). Correlation of an epigenetic mitotic clock with cancer risk. Genome Biol.

[63] Zbiec-Piekarska R, Spolnicka M, Kupiec T, Parys-Proszek A, Makowska Z, Paleczka A, Kucharczyk K, Ploski R, Branicki W (2015). Development of a forensically useful age prediction method based on DNA methylation analysis. Forensic Sci Int Genet.

[64] Zeileis A (2004). Econometric computing with hc and hac covariance matrix estimators. J Stat Softw.

[65] Zheng C, Berger NA, Li L, Xu R (2020). Epigenetic age acceleration and clinical outcomes in gliomas. PLoS ONE.

[66] Zou H, Hastie T (2005). Regularization and variable selection via the Elastic Net. J R Stat Soc Ser B (Stat Methodol).

