# Supplementary material for: DNA methylation-based age acceleration observed in IDH wild-type glioblastoma is associated with better outcome—including in elderly patients
Source: Acta Neuropathol Commun. 2022 Mar 24;10:39. doi: 10.1186/s40478-022-01344-5 (PMC8944086; doi:10.1186/s40478-022-01344-5)
Supplement: Supplementary file 1 — Additional file. 1. Table S1.Description of full EORTC/NCIC & LN-Pilot and Nordic datasets. Table S2. DNA methylation associated with patient age. Table S3. Associations of methylation based entropy by genomic region and GBM classification. Table S4. Functional CpGs located on DDR genes, associated with Age Acceleration. Table S5. Functional CpGs located on DDR genes, associated with GBM classification. Fig. S1 Methylation-based classification of tumors of all patients from EORTC/NCIC & LN-Pilot and Nordic studies. Fig. S2 Impact of sample purity on measures of DNAm age acceleration, and HM entropy. Fig. S3 Pathways associated with functional methylation related to DNAm age acceleration and GBM classification. Fig. S4 CDKN2A CNV and DNAm age acceleration. [file 40478_2022_1344_MOESM1_ESM.pdf]

Additional File 1

Supplementary Tables S1-S5

- **Table S1.** Description of full EORTC/NCIC & LN-Pilot and Nordic datasets.
- **Table S2.** DNA methylation associated with patient age.
- **Table S3.** Associations of methylation based entropy by genomic region and GBM classification.
- **Table S4.** Functional CpGs located on DDR genes, associated with Age Acceleration
- **Table S5.** Functional CpGs located on DDR genes, associated with GBM classification.

Supplementary Figures S1-S3

- **Figure S1.** Methylation-based classification of tumors of all patients from EORTC/NCIC & LN-Pilot and Nordic studies.
- **Figure S2**. Impact of sample purity on measures of DNAm age acceleration, and HM entropy
- **Figure S3.** Pathways associated with functional methylation related to DNAm age acceleration and GBM classification.
- **Figure S4.** *CDKN2A* CNV and DNAm age acceleration

Table S1: Description of full EORTC/NCIC & LN-Pilot and Nordic datasets

	**EORTC/NCIC & LN-Pilot (N = 219)**	**Nordic (N = 116)**
**Age [years]**		
minimum	25.00	60.05
median (IQR)	53.50 (48.00, 60.00)	70.41 (66.51, 74.09)
mean (SD)	52.86 ± 9.77	70.36 ± 4.80
maximum	70.00	83.06
Unknown/Missing	1 (0.46%)	0 (0.00%)
**DNAm age [years]**		
minimum	31.18	51.90
median (IQR)	90.12 (71.47, 108.11)	103.19 (83.53, 123.92)
mean (SD)	91.26 ± 27.23	105.47 ± 25.88
maximum	167.65	180.46
**Accel [years]**		
minimum	-24.40	-12.07
median (IQR)	35.66 (21.11, 54.72)	32.24 (15.48, 52.18)
mean, SD	38.37 ± 26.03	35.11 ± 25.26
maximum	107.34	108.46
Unknown/Missing	1 (0.46%)	0 (0.00%)
**Sex** (%)		
F	78 (36)	43 (37)
M	141 (64)	73 (63)
**WHO Performance Score (%)**		
0	95 (44)	29 (25)
1	107 (49)	61 (53)
2	16 (7)	22 (19)
3	0 (0)	4 (3)
Unknown/Missing	1 (0.46%)	0 (0.00%)
**MGMTscore**		
minimum	-10.48	-4.09
median (IQR)	-0.73 (-2.68, 3.80)	-0.93 (-2.66, 3.70)
mean, SD	0.29 ± 3.40	0.31 ± 3.33
maximum	7.35	8.16
**MGMT meth status (%)**		
M	107 (49)	56 (48)
U	112 (51)	60 (52)
**hCIMP** (%)		
cimp	15 (7)	1 (1)
noncimp	199 (93)	115 (99)
Unknown/Missing	5 (2.28%)	0 (0.00%)

Continued next page

Table S1, continued from previous page

	**EORTC/NCIC & LN-Pilot, N=219**	**Nordic, N=116**
***MNP classification**		
A_IDH	2 (1)	0 (0)
A_IDH_HG	11 (5)	1 (1)
ANA_PA	1 (0)	0 (0)
CONTR_HEMI	1 (0)	0 (0)
CONTR_INFLAM	3 (1)	7 (6)
CONTR_REACT	1 (0)	0 (0)
CONTR_WM	1 (0)	0 (0)
DMG_K27	1 (0)	0 (0)
EPN_RELA	1 (0)	0 (0)
GBM_G34	1 (0)	0 (0)
GBM_MES	69 (32)	43 (37)
GBM_MID	2 (1)	2 (2)
GBM_MYCN	1 (0)	0 (0)
GBM_RTK_I	32 (15)	20 (17)
GBM_RTK_II	85 (39)	42 (36)
O_IDH	2 (1)	0 (0)
PLEX_PED_B	2 (1)	1 (1)
PXA	3 (1)	0 (0)
**Study (%)**		
EORTC/NCIC & LN-Pilot	219 (100)	0 (0)
Nordic	0 (0)	116 (100)

*MNP classification, Methylation-based classification of CNS tumors, Molecular Neuropathology

Abbreviations: Accel, DNA methylation age acceleration; DNAm age, DNA methylation age**;** hcimp; human CpG island methylation phenotype; QR. interquartile range; SD, standard deviation

Table S2. DNA methylation associated with patient age

ProbeID	Symbol	Slope (b)	R-squared	F-value	Pr(>F)	P-Bonferroni	Location type	Relation Island	*Element Type	Assoc Accel	Assoc Class	Corr (Expr Methyl)	p-value	P-Bonf
cg03514351	LEPR	0.044365	0.05471527	34.3242168	7.7391E-09	0.00279959	gene; prom	Island	Prom/Enh	**TRUE**	**TRUE**			
cg08816037	IFT80; TRIM59	0.03865695	0.05110595	31.9380543	2.4741E-08	0.00894991	promoter	Island	Prom/Enh	**TRUE**	FALSE	-0.56471	0.000511	0.028469
cg16867657	ELOVL2-AS1; ELOVL2	0.0238864	0.05436931	34.0947058	8.6519E-09	0.0031298	promoter	Island	Prom/Enh	**TRUE**	**TRUE**			
cg06335867	NXPH1	0.04966675	0.06025499	38.0222419	1.2941E-09	0.00046815	gene	Island		**TRUE**	**TRUE**			
cg12597389	NXPH1	0.04007597	0.05428946	34.0417571	8.8775E-09	0.00321137	gene	Island		**TRUE**	FALSE			
cg12744812	MNX1-AS1	0.02823639	0.04488323	27.8664939	1.8264E-07	0.06606765	extend	Island		FALSE	**TRUE**			
cg16197925	FEZF1-AS1; FEZF1	0.0279542	0.04686293	29.1560574	9.6744E-08	0.03499674	promoter	Island	Prom/Enh	**TRUE**	FALSE			
cg26170604	NXPH1	0.04976372	0.04570145	28.3988245	1.4046E-07	0.05081095	gene	Island	Enhancer	FALSE	**TRUE**			
cg26818735	TWIST1	0.05287926	0.04393627	27.2515378	2.4747E-07	0.08952102	promoter	Island	Prom/Enh	**TRUE**	FALSE	-0.60784	0.000147	0.013656
cg03323636	NRIP3	0.04335599	0.05311561	33.2644194	1.2957E-08	0.00468715	promoter	Island	Prom/Enh	**TRUE**	FALSE	-0.59272	0.000232	0.017835
cg23132624	KL	0.04874967	0.0545758	34.2316671	8.095E-09	0.00292831	promoter	Island	Prom/Enh	**TRUE**	FALSE			
cg02970384	ACTA1	0.02676012	0.05000415	31.2132523	3.5262E-08	0.01275585	gene	Island	Prom/Enh	**TRUE**	FALSE			
cg06527052	TMEM167B	0.00617915	0.04850109	30.2271997	5.7163E-08	0.02067838	promoter	S_Shore	Prom/Enh	FALSE	FALSE			
cg23704082	IFT80; TRIM59	0.04009039	0.04700147	29.2464994	9.2535E-08	0.03347408	promoter	Island	Prom/Enh	**TRUE**	FALSE			
cg06470822		0.03211127	0.0456596	28.3715794	1.4236E-07	0.05149791	intergenic	Island		**TRUE**	**TRUE**			
cg20974724	FEZF1-AS1; FEZF1	0.03674432	0.0519421	32.4892225	1.8905E-08	0.00683876	promoter	Island	Prom/Enh	**TRUE**	FALSE			
cg25098077	PDLIM1	0.03457268	0.04716098	29.3506692	8.7915E-08	0.03180264	promoter	Island	Prom/Enh	**TRUE**	FALSE			
cg01994205	RNF219-AS1;POU4F1	0.04504875	0.04792986	29.8532717	6.8677E-08	0.0248437	gene; prom	Island	Prom/Enh	**TRUE**	FALSE			
cg02796545	KL	0.0353594	0.05429518	34.0455517	8.8611E-09	0.00320546	promoter	Island	Prom/Enh	**TRUE**	FALSE			

*genehancer 2.0

Table S3. Associations of methylation based entropy by genomic region and GBM classification.

Characteristic	Prom	CpG-Island relationship	MES mean (sd)	RTK I mean (sd)	RTK II mean (sd)	GBM classification F-value	GBM classification Pr(>F)	Study F-value	Study Pr(>F)	GBM classification x Study F-value	GBM classification x Study Pr(>F)
NoneIsland	no	CpGs-Island	0.557 (0.029)	0.567 (0.029)	0.55 (0.03)	6.207053	0.00214944	0.05208871	0.984304	0.2322369	0.966069364
NoneN_Shelf	no	N_Shelf	0.639 (0.043)	0.685 (0.038)	0.629 (0.042)	39.4388337	8.4277E-17	0.30650644	0.8207	1.2374211	0.285187616
NoneN_Shore	no	N_shore	0.653 (0.034)	0.675 (0.032)	0.638 (0.035)	24.9416396	4.0024E-11	0.12473897	0.945475	0.7216483	0.632292687
NoneOpenSea	no	Open Sea	0.643 (0.039)	0.673 (0.036)	0.625 (0.039)	30.9767708	1.6161E-13	0.20681642	0.891689	0.9450378	0.462079214
NoneS_Shelf	no	S_Shelf	0.637 (0.043)	0.683 (0.038)	0.627 (0.042)	38.2902463	2.3248E-16	0.29527014	0.82883	1.1771564	0.316748706
NoneS_Shore	no	S_shore	0.64 (0.034)	0.66 (0.032)	0.624 (0.034)	23.592329	1.3922E-10	0.11733844	0.949928	0.5937028	0.735497569
PromIsland	yes	CpGs-Island	0.362 (0.032)	0.361 (0.03)	0.363 (0.029)	0.08626142	0.91736597	0.14667742	0.93181	1.8152771	0.093836959
PromN_Shelf	yes	N_Shelf	0.662 (0.035)	0.686 (0.033)	0.644 (0.036)	28.3923689	1.6908E-12	0.13803558	0.93727	1.1699905	0.320671299
PromN_Shore	yes	N_shore	0.55 (0.026)	0.545 (0.027)	0.535 (0.025)	6.71057346	0.00131324	0.04780861	0.986145	0.3061348	0.933792612
PromOpenSea	yes	Open Sea	0.648 (0.034)	0.666 (0.032)	0.63 (0.034)	22.4423925	4.0449E-10	0.11010916	0.954191	0.984101	0.435052346
PromS_Shelf	yes	S_Shelf	0.639 (0.037)	0.67 (0.033)	0.622 (0.037)	35.6277052	2.4762E-15	0.20068678	0.895914	1.1349293	0.340387478
PromS_Shore	yes	S_shore	0.548 (0.026)	0.543 (0.027)	0.532 (0.025)	7.39443155	0.00067343	0.05563908	0.982727	0.27667	0.947916261
Global HME	no	none	0.556 (0.029)	0.571 (0.027)	0.545 (0.029)	18.5570792	1.5293E-08	0.02850008	0.993511	0.3939696	0.883032642

Association tests with GBM classification, study origin and the interaction between these both variables for the entropy metrics based on DNA methylation Entropy (HME) and stratified by the Island regions (CpG islands, shores, shelves or open sea; N, north; S, south) and promoter location status (promoter or not in promoter). The linear models are compared by Wald's test using the sandwich covariance matrix (type HC3) and F-statistic to compensate heteroskedasticity.

Table S4: Description of the CpGs located in DDR genes, associated with DNAmeth Age Acceleration and considered as functional.

ProbeID	EntrezID	Symbol	RnaseqID	Correlation Expr vs Methyl			Age		Age acceleration		Classification
				cor	p.value	padj	slope	R2	slope	R2	R2
cg05382305	164045	HFM1	HFM1|164045	-0.4857	0.0034	0.0938	-0.0027	0.0010	0.0082	0.0417	0.0035
cg23032045	164045	HFM1	HFM1|164045	-0.5440	0.0009	0.0479	-0.0123	0.0090	0.0128	0.0462	0.0119
cg15091337	56655	POLE4	POLE4|56655	-0.6751	0.0000	0.0034	0.0019	0.0002	0.0163	0.0780	0.0216
cg12290764	56655	POLE4	POLE4|56655	-0.7347	0.0000	0.0009	0.0015	0.0001	0.0243	0.1361	0.0253
cg02058002	56655	POLE4	POLE4|56655	-0.7387	0.0000	0.0009	0.0001	0.0000	0.0243	0.1157	0.0198
cg02307033	56655	POLE4	POLE4|56655	-0.7894	0.0000	0.0007	0.0036	0.0005	0.0267	0.1150	0.0270
cg20919922	56655	POLE4	POLE4|56655	-0.7459	0.0000	0.0009	0.0039	0.0003	0.0348	0.1180	0.0260
cg12696259	56655	POLE4	POLE4|56655	-0.6499	0.0000	0.0065	0.0178	0.0088	0.0314	0.1285	0.0224
cg13690354	56655	POLE4	POLE4|56655	-0.7045	0.0000	0.0017	0.0098	0.0027	0.0283	0.1042	0.0347
cg05778415	56655	POLE4	POLE4|56655	-0.6120	0.0001	0.0153	0.0078	0.0021	0.0288	0.1352	0.0151
cg20142358	57599	WDR48	WDR48|57599	-0.4908	0.0031	0.0882	0.0032	0.0041	0.0045	0.0389	0.0181
cg15438497	6596	HLTF	HLTF|6596	-0.4812	0.0038	0.0982	-0.0028	0.0008	0.0139	0.0956	0.0515
cg03678609	6596	HLTF	HLTF|6596	-0.4980	0.0026	0.0789	-0.0025	0.0004	0.0212	0.1229	0.0827
cg07562918	1029	CDKN2A	CDKN2A|1029	-0.6429	0.0000	0.0076	0.0043	0.0038	0.0096	0.0873	0.1398
cg14194875	4255	MGMT	MGMT|4255	-0.7151	0.0000	0.0014	0.0188	0.0107	0.0160	0.0365	0.0429
cg00618725	4255	MGMT	MGMT|4255	-0.6258	0.0001	0.0113	0.0213	0.0165	0.0145	0.0364	0.0373
cg12434587	4255	MGMT	MGMT|4255	-0.7983	0.0000	0.0007	0.0178	0.0052	0.0257	0.0513	0.0188
cg02802904	4255	MGMT	MGMT|4255	-0.5109	0.0020	0.0701	0.0148	0.0099	0.0128	0.0345	0.0165
cg12981137	4255	MGMT	MGMT|4255	-0.7269	0.0000	0.0010	0.0271	0.0120	0.0235	0.0429	0.0415
cg02941816	4255	MGMT	MGMT|4255	-0.6880	0.0000	0.0025	0.0171	0.0114	0.0132	0.0324	0.0282
cg20808578	1454	CSNK1E	CSNK1E|1454	-0.5711	0.0004	0.0323	0.0011	0.0002	0.0064	0.0331	0.0001
cg22884516	27127	SMC1B	SMC1B|27127	-0.5134	0.0018	0.0687	0.0212	0.0117	0.0313	0.1193	0.0517

Table S5. Functional CpGs located in DDR genes, associated with GBM classification.

ProbeID	EntrezID	Symbol	RnaseqID	Correlation Expr vs Methylation			Age		Age acceleration		GBM classification
				corr	p.value	padj	slope	R2	slope	R2	R2
cg15438497	6596	HLTF	HLTF|6596	-0.4812	0.0038	0.0982	-0.0028	0.0008	0.0139	0.0956	0.0515
cg03678609	6596	HLTF	HLTF|6596	-0.4980	0.0026	0.0789	-0.0025	0.0004	0.0212	0.1229	0.0827
cg24183261	2138	EYA1	EYA1|2138	-0.5165	0.0017	0.0662	-0.0004	0.0000	0.0023	0.0057	0.0456
cg13043862	2138	EYA1	EYA1|2138	-0.5174	0.0017	0.0662	0.0026	0.0018	0.0022	0.0061	0.0389
cg13601799	1029	CDKN2A	CDKN2A|1029	-0.7384	0.0000	0.0009	0.0086	0.0116	0.0024	0.0042	0.0831
cg03079681	1029	CDKN2A	CDKN2A|1029	-0.6958	0.0000	0.0020	0.0059	0.0102	0.0014	0.0026	0.0927
cg07562918	1029	CDKN2A	CDKN2A|1029	-0.6429	0.0000	0.0076	0.0043	0.0038	0.0096	0.0873	0.1398
cg10848754	1029	CDKN2A	CDKN2A|1029	-0.7062	0.0000	0.0017	0.0125	0.0208	0.0061	0.0231	0.0976
cg14430974	1029	CDKN2A	CDKN2A|1029	-0.7017	0.0000	0.0017	0.0143	0.0243	0.0067	0.0252	0.0969
cg14194875	4255	MGMT	MGMT|4255	-0.7151	0.0000	0.0014	0.0188	0.0107	0.0160	0.0365	0.0429
cg00618725	4255	MGMT	MGMT|4255	-0.6258	0.0001	0.0113	0.0213	0.0165	0.0145	0.0364	0.0373
cg12981137	4255	MGMT	MGMT|4255	-0.7269	0.0000	0.0010	0.0271	0.0120	0.0235	0.0429	0.0415
cg25419628	9937	DCLRE1A	DCLRE1A|9937	-0.5535	0.0007	0.0432	0.0069	0.0138	0.0042	0.0248	0.0591
cg03727700	9937	DCLRE1A	DCLRE1A|9937	-0.5473	0.0008	0.0470	0.0027	0.0043	0.0030	0.0248	0.0429
cg18787244	9937	DCLRE1A	DCLRE1A|9937	-0.5947	0.0002	0.0215	0.0021	0.0033	0.0020	0.0140	0.0443
cg03817911	493861	EID3	EID3|493861	-0.5042	0.0023	0.0743	0.0019	0.0002	0.0001	0.0000	0.1450
cg09096528	1019	CDK4	CDK4|1019	-0.6812	0.0000	0.0030	0.0036	0.0059	0.0000	0.0000	0.0424
cg11869215	328	APEX1	APEX1|328	-0.5535	0.0007	0.0432	0.0042	0.0076	-0.0025	0.0127	0.0428
cg06755612	57697	FANCM	FANCM|57697	-0.4958	0.0028	0.0795	0.0008	0.0003	-0.0008	0.0015	0.0353
cg25564554	8846	ALKBH1	ALKBH1|8846	-0.5120	0.0019	0.0690	0.0043	0.0097	-0.0013	0.0039	0.0433
cg13367381	10054	UBA2	UBA2|10054	-0.5521	0.0007	0.0432	0.0022	0.0050	-0.0013	0.0087	0.0359
cg22884516	27127	SMC1B	SMC1B|27127	-0.5134	0.0018	0.0687	0.0212	0.0117	0.0313	0.1193	0.0517

Supplementary Figures

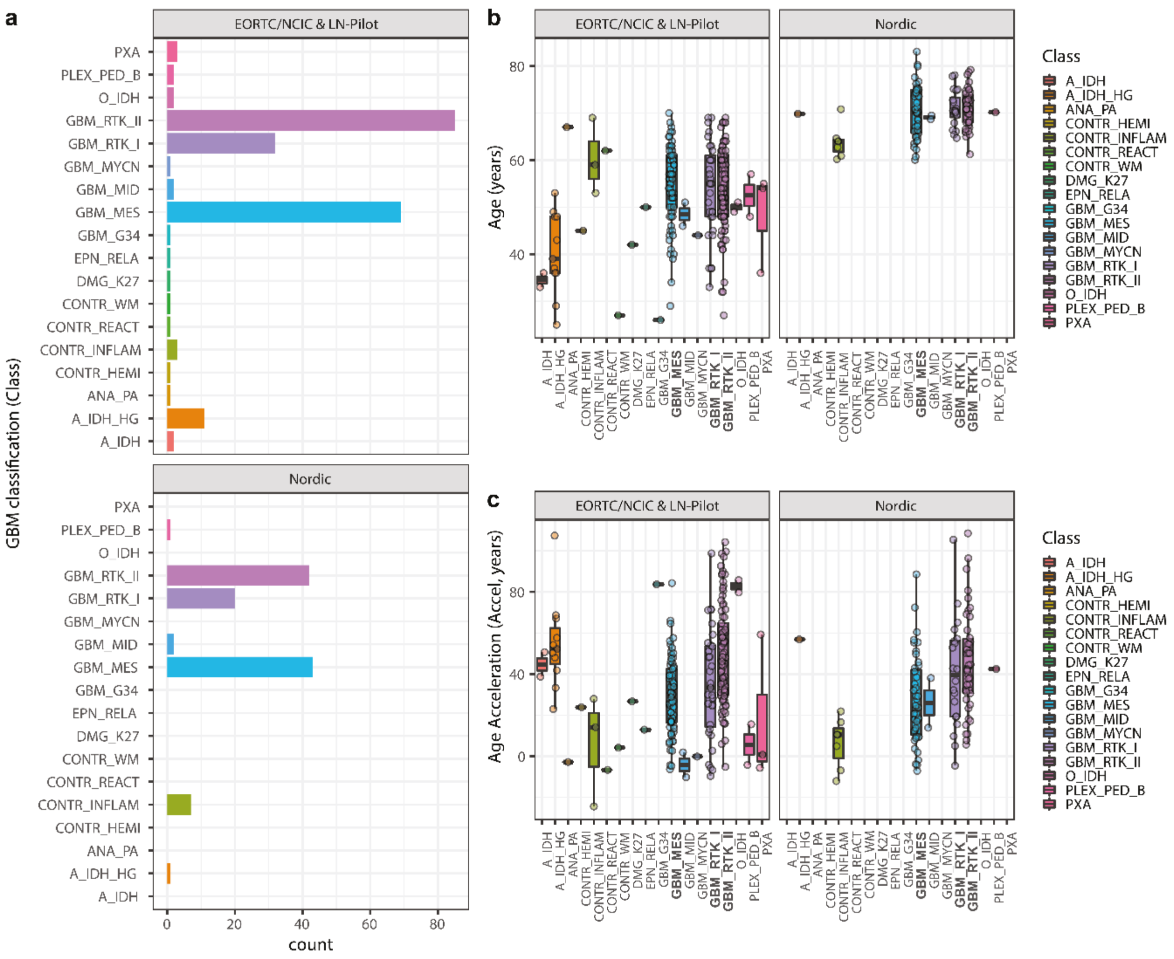

Fig. S1 Methylation-based classification of tumors of all patients from EORTC/NCIC & LN-Pilot and Nordic studies. (**a**) The number of patients by methylation-based classification subtype is visualized for patients in the EORTC/NCIC & LN-Pilot and Nordic studies. The variables age (**b**) and DNAm age acceleration (**c**) are illustrated in function of the molecular subgroups and the study origin.

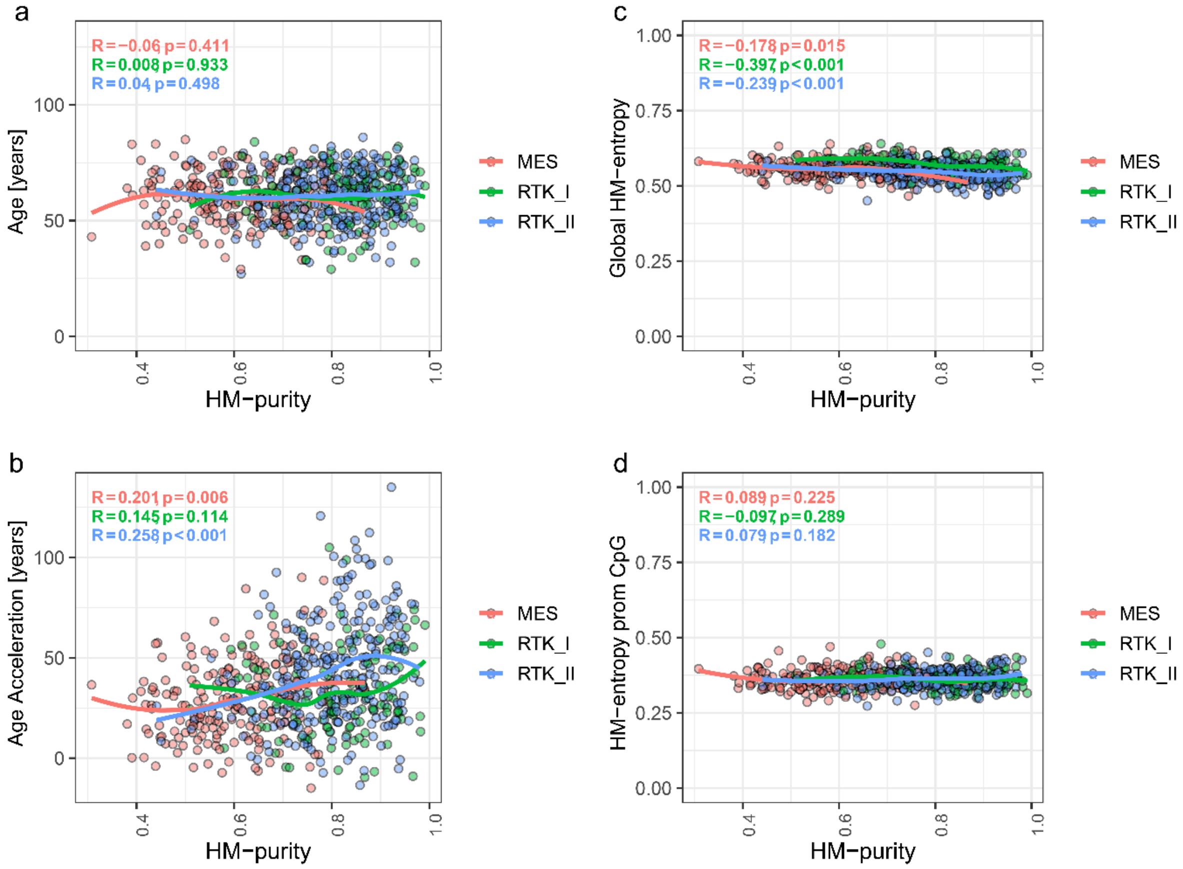

Fig. S2 Impact of sample purity on measures of DNAm age acceleration and HM entropy. Association of the patients' age **a**, age acceleration **b**, global HM-entropy (HM-entropy) **c**, and HM-entropy in the promoter region **d,** with purity estimated by DNA methylation (HM-purity), stratified by GBM subtype. The association trend between HM-purity and the four variables is given by loess regression and the Spearman's correlation, provided for each variable stratified by GBM subtype. No effect of purity was observed for patient age, and HM-entropy in the promoter region, while a weak effect was observed for global HM-entropy and age acceleration. A significant association of purity and GBM subtype is shown in Fig. 1**e**.

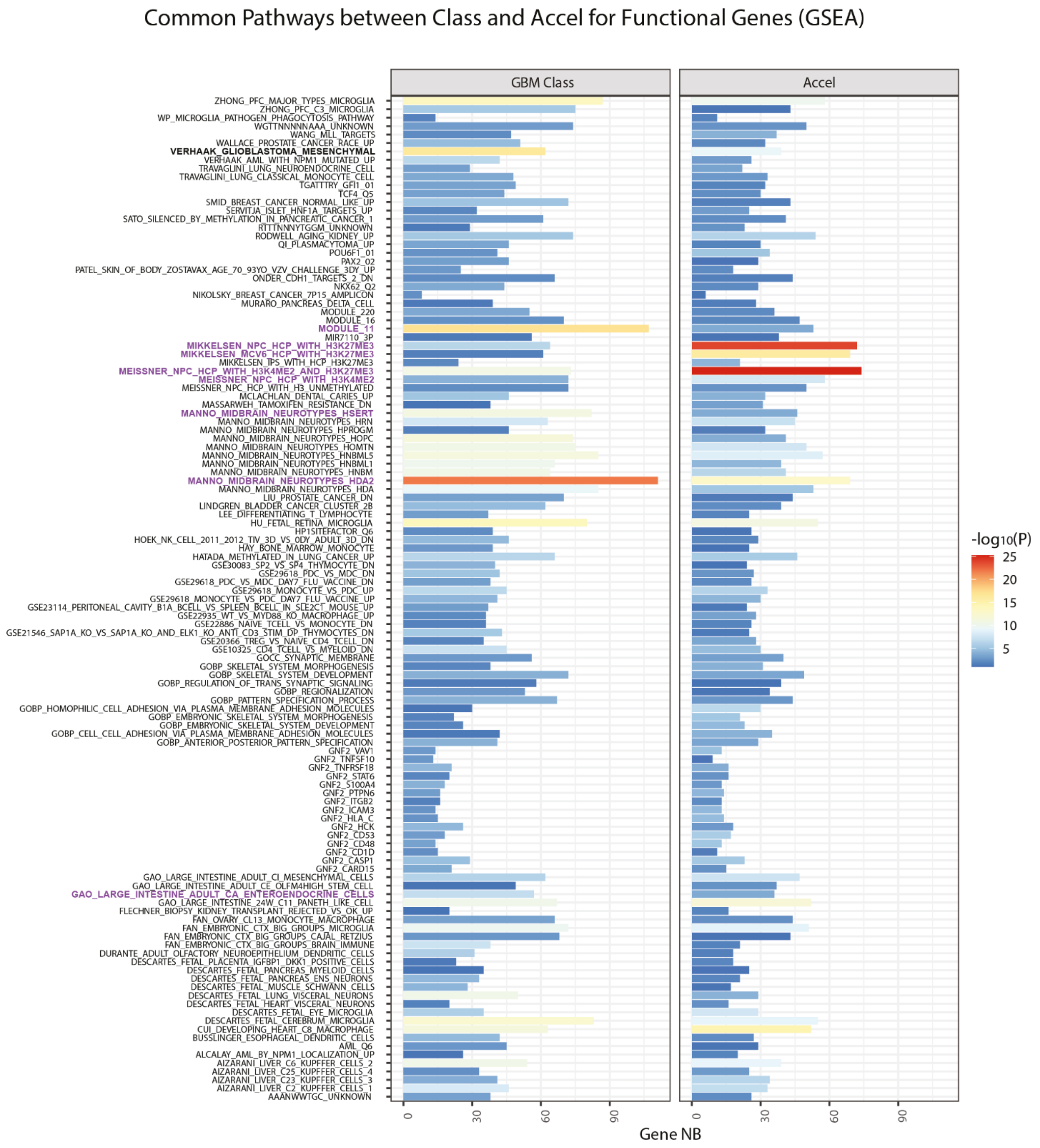

Fig. S3 Pathways associated with functional methylation related to DNAm age acceleration and GBM classification. Gene set enrichment analysis (GSEA) was established for functional candidate CpG-probes located in gene promoters associated with age, DNAm age acceleration, and GBM classification. Functional methylated positions were defined as functional, when the correlation coefficient was inferior to -0.3 (negative effect of methylation on gene expression) and the q-value was less than 0.1. No significant "functional" pathways were detected for patients' age. The "functional" pathways associated with DNAm age accel (n=167) mostly (119, 71%) overlapped with those associated with classification (n=294). The list retained, comprises selected pathways significantly enriched for both, DNAm age

acceleration and GBM classification. The number (NB) of genes per gene set is indicated and the p-value is represented by the color code. The gene sets overlapping with those identified significant for genome wide DNA methylation (Fig. 3**d**) for classification and DNAm age acceleration are highlighted in color. Of note, the Verhaak signature for mesenchymal GBM is part of this list, marked in bold. The pathways are listed in alphabetical order. GSEA investigation is based on the MSigDB database.

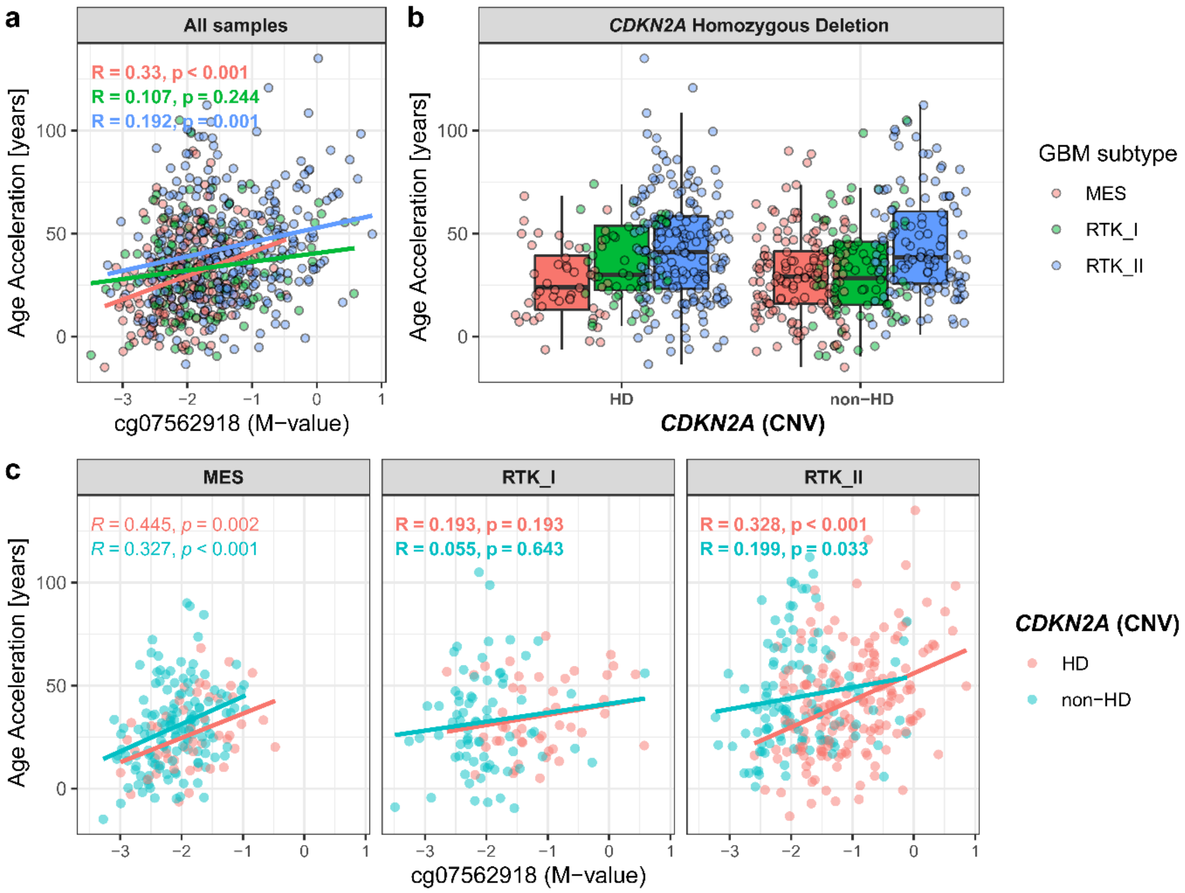

Fig. S4 *CDKN2A* methylation, homozygous deletion and Age Acceleration. Correlation of methylation and DNAm age acceleration in all samples, stratified by GBM subtype **a**. Description of association of age acceleration with homozygous deletion status of *CDKN2A* (HD or non-HD) and the CpG-probe cg07562918 (Wilcoxon test, p=0.163) stratified by GBM subtype **b**. The representation of DNAm age acceleration in function of the M-value of the probe cg07562918, stratified by GBM group and *CDKN2A* homozygous deletion status **c**.
